# Supplementary material for: In situ generation of micrometer-sized tumor cell-derived vesicles as autologous cancer vaccines for boosting systemic immune responses
Source: Nat Commun. 2022 Nov 1;13:6534. doi: 10.1038/s41467-022-33831-7 (PMC9626595; doi:10.1038/s41467-022-33831-7)
Supplement: Supplementary file 1 — Supplementary Information [file 41467_2022_33831_MOESM1_ESM.pdf]

Supplementary Information

In situ generation of micrometer-sized tumor cell-derived vesicles as autologous cancer vaccines for boosting systemic immune responses

Yuxin Guo, Shao-Zhe Wang, Xinping Zhang, Hao-Ran Jia, Ya-Xuan Zhu, Xiaodong Zhang, Ge Gao, Yao-Wen Jiang, Chengcheng Li, Xiaokai Chen, Shun-Yu Wu, Yi Liu, and Fu-Gen Wu*

State Key Laboratory of Bioelectronics, School of Biological Science and Medical Engineering, Southeast University, 2 Sipailou Road, Nanjing 210096, P. R. China

*Corresponding author

E-mail address: wufg@seu.edu.cn

Supplementary Figures

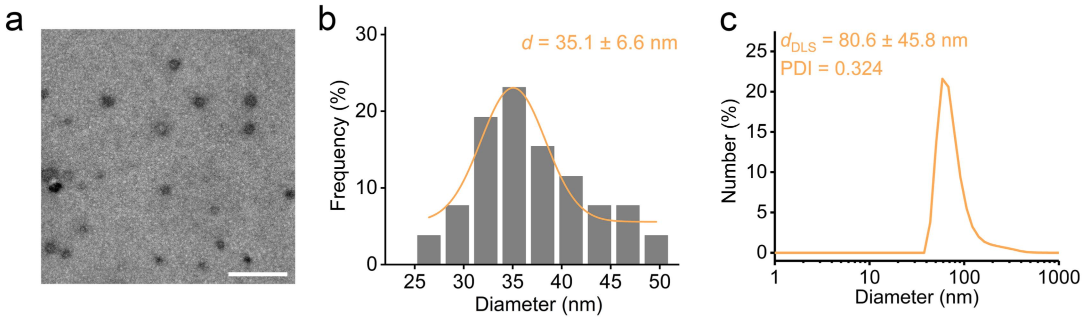

Supplementary Figure 1. (**a**) TEM image and (**b**) corresponding size distribution histogram of Den−DOX−Apa (DDA). Scale bar: 300 nm. The experiment was performed twice with similar results. (**c**) Hydrodynamic diameter of DDA nanoparticles (NPs).

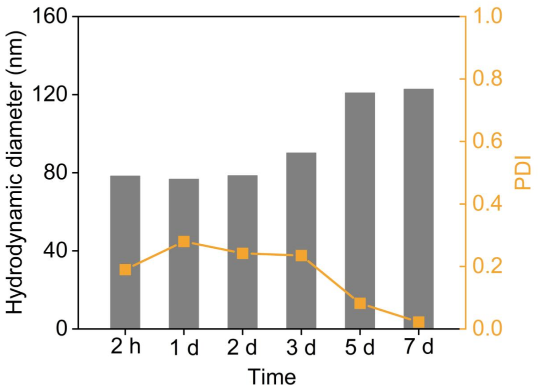

Supplementary Figure 2. Hydrodynamic diameters of HDDA NBs measured by DLS at indicated time points. The experiment was performed twice with similar results.

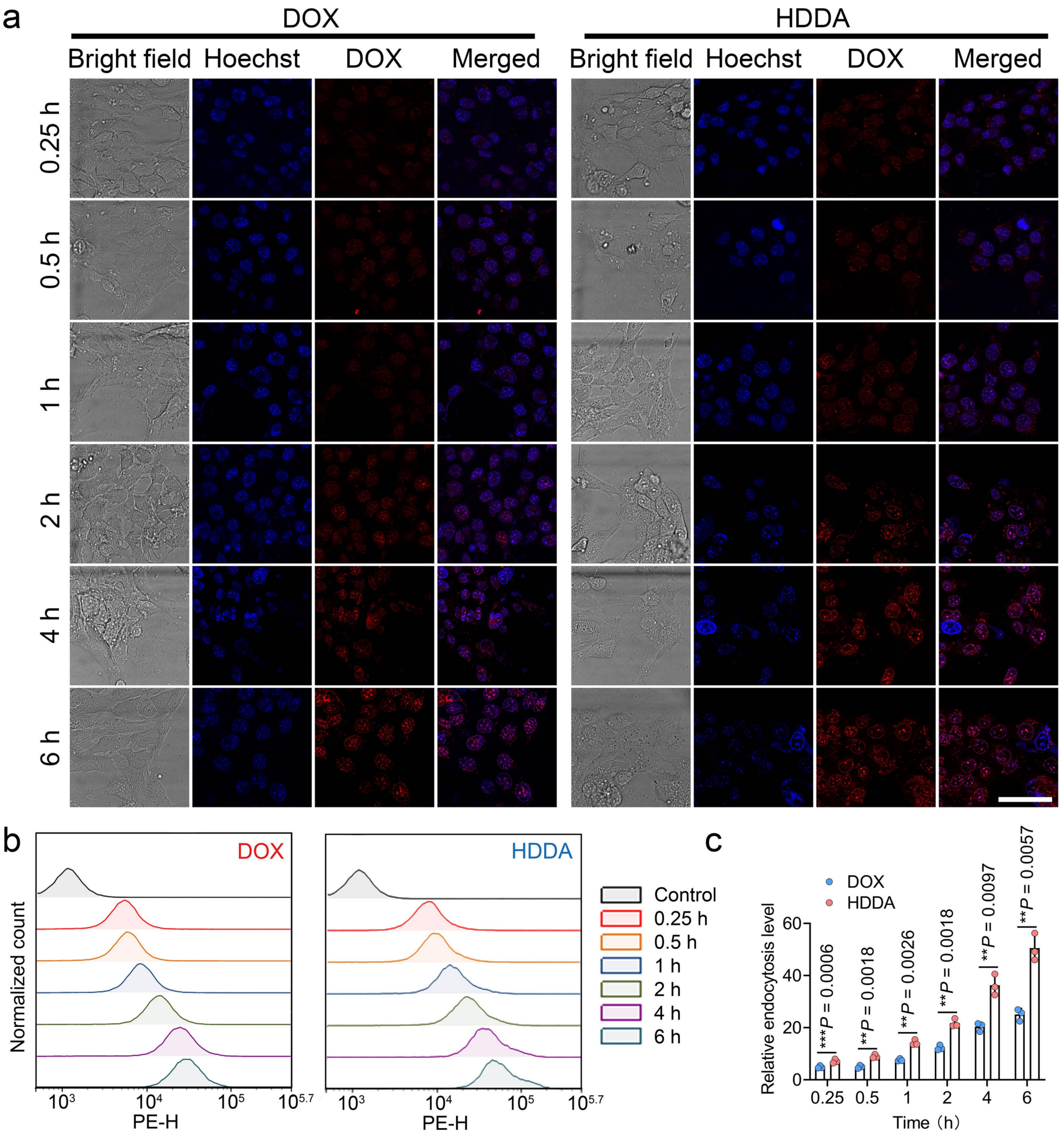

Supplementary Figure 3. (**a**) Confocal microscopic images of the 4T1 cells after incubation with DOX or HDDA for different time periods. Before imaging, the cell nuclei were stained with Hoechst. Scale bar: 50 μm. (**b**) Flow cytometric results showing the DOX fluorescence intensities in the 4T1 cells before (control) and after incubation with DOX or HDDA for different time periods, and (**c**) relative endocytosis levels of the cells subjected to the DOX or HDDA treatments derived from the flow cytometric data in (**b**). The cellular autofluorescence intensity in the control group was set as 1. The DOX concentration of DOX and HDDA was 1.8 μg/mL. Data are presented as mean ± SD. n = 3 biologically independent samples per group. $**P < 0.01$, $***P < 0.001$. Statistical significance in (**c**) was calculated via two-tailed Student's t-test. The experiment was performed twice with similar results.

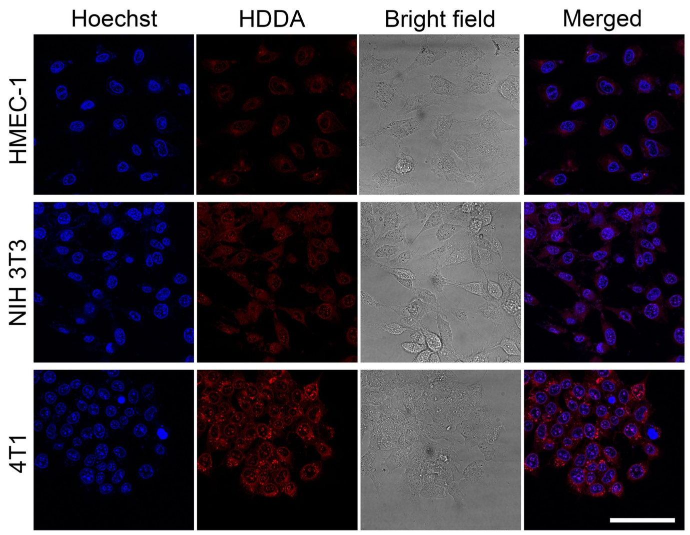

Supplementary Figure 4. Confocal microscopic images of the HMEC-1, NIH 3T3, and 4T1 cells after incubation with HDDA for 2 h. Before imaging, the cell nuclei were stained with Hoechst. Scale bar: 75 μm. The DOX concentration in HDDA was 3.6 μg/mL. The experiment was performed twice with similar results.

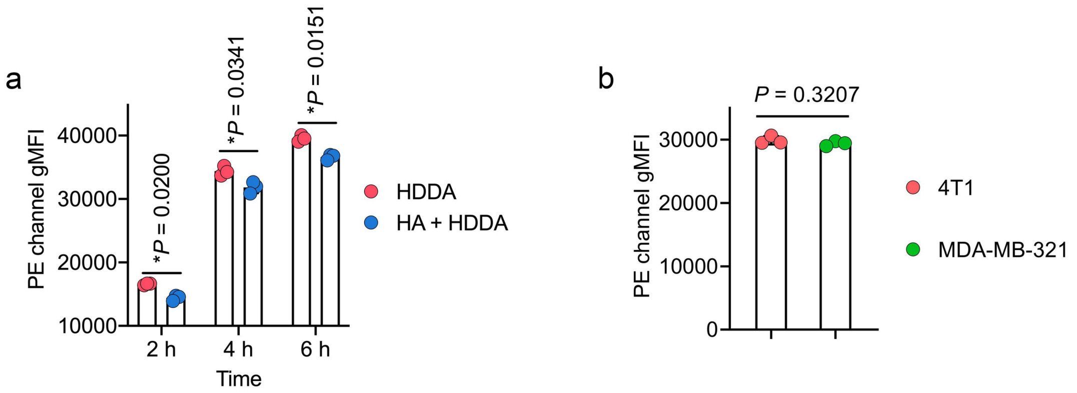

Supplementary Figure 5. (**a**) Flow cytometric results showing the DOX fluorescence intensities in the 4T1 cells after incubation with HDDA NBs (DOX concentration: 3.6 μg/mL) for different time periods as indicated. For the HA + HDDA group, the 4T1 cells were pretreated with HA (1 mg/mL) before incubating with the HDDA NBs. $^*P < 0.05$. (**b**) Flow cytometric results showing the DOX fluorescence intensities in the 4T1 and MDA-MB-231 cells after incubation with HDDA NBs (DOX concentration: 3.6 μg/mL) for 3 h. gMFI: geometric mean fluorescence intensity. Data are presented as mean ± SD. n = 3 biologically independent samples per group. Statistical significance was calculated via two-tailed Student's t-test.

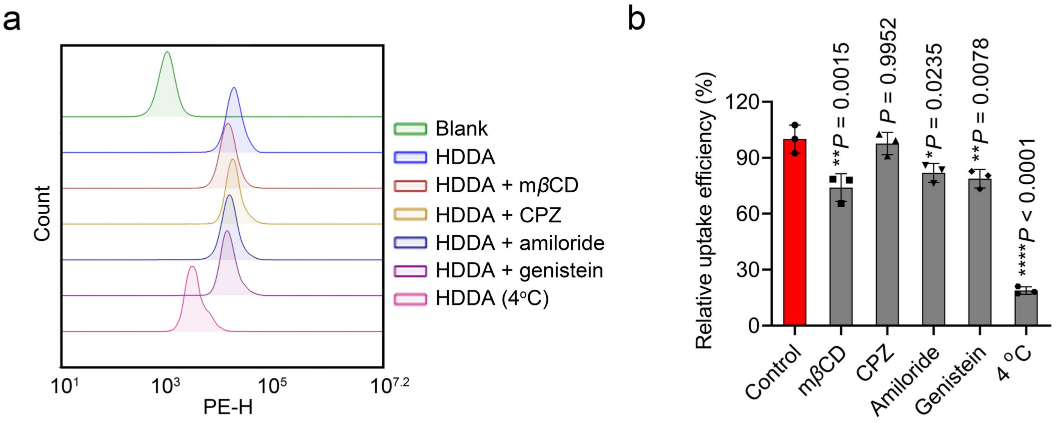

Supplementary Figure 6. (**a**) Flow cytometric results and (**b**) corresponding statistic results showing the DOX fluorescence intensities in the 4T1 cells after incubation with HDDA under different conditions as indicated. The 4T1 cells without drug treatment were set as the blank group, and the HDDA-treated 4T1 cells were set as the control group in (**b**). The DOX concentration in HDDA was 3.6 μg/mL. Data are presented as mean ± SD. $n = 3$ biologically independent samples per group. *$P < 0.05$, **$P < 0.01$, ****$P < 0.0001$. Statistical significance in **b** was calculated via one-way ANOVA with a Tukey's post-hoc test.

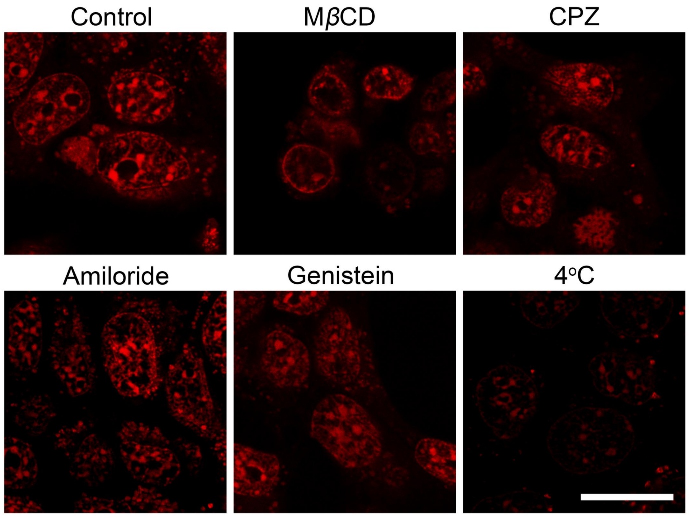

Supplementary Figure 7. Confocal fluorescence images of 4T1 cells after incubation with HDDA under different conditions as indicated. Scale bar: 20 μm. The DOX concentration in HDDA was 3.6 μg/mL. The experiment was performed three times with similar results.

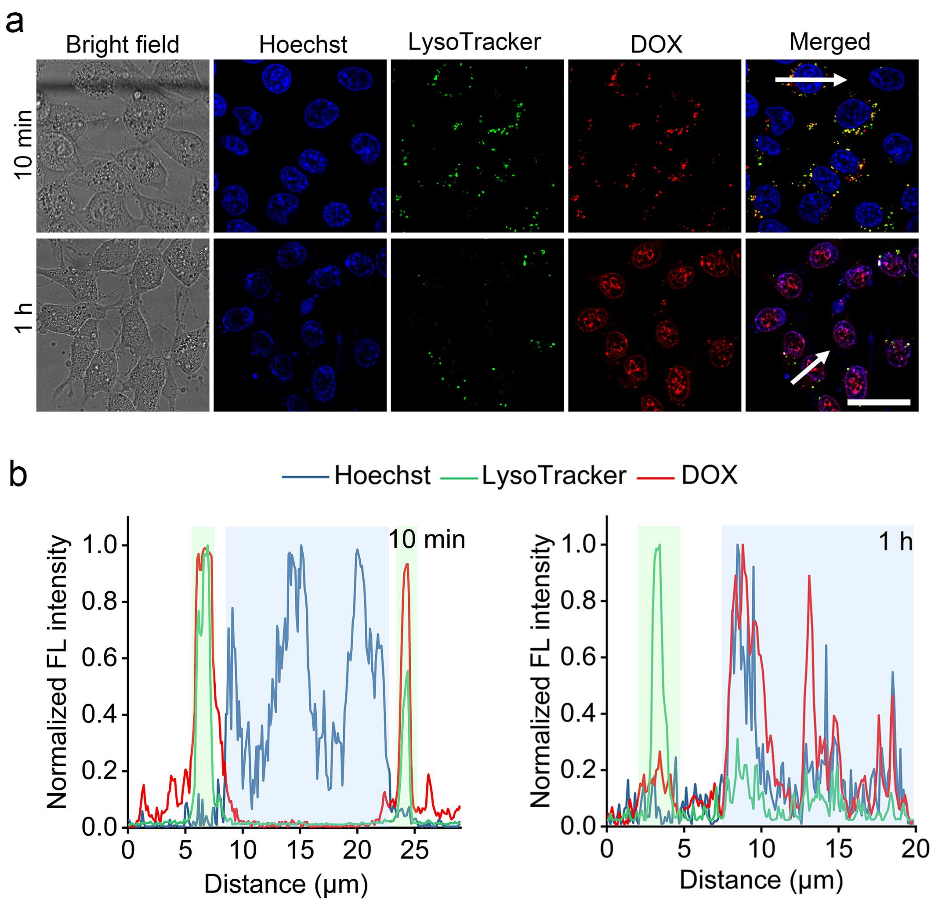

Supplementary Figure 8. (**a**) Confocal microscopic images of the HDDA-treated 4T1 cells at 10 min and 1 h. The nuclei and lysosomes were visualized by Hoechst 33342 (abbreviated as Hoechst) and LysoTracker Green (abbreviated as LysoTracker), respectively. Scale bar: 30 μm. (**b**) Normalized line-scan fluorescence intensities of the white arrow-marked positions in (**a**). The blue- and green-shaded areas indicate the nuclear and lysosomal positions, respectively. The DOX concentration was fixed at 3.6 μg/mL. The experiment was performed three times with similar results.

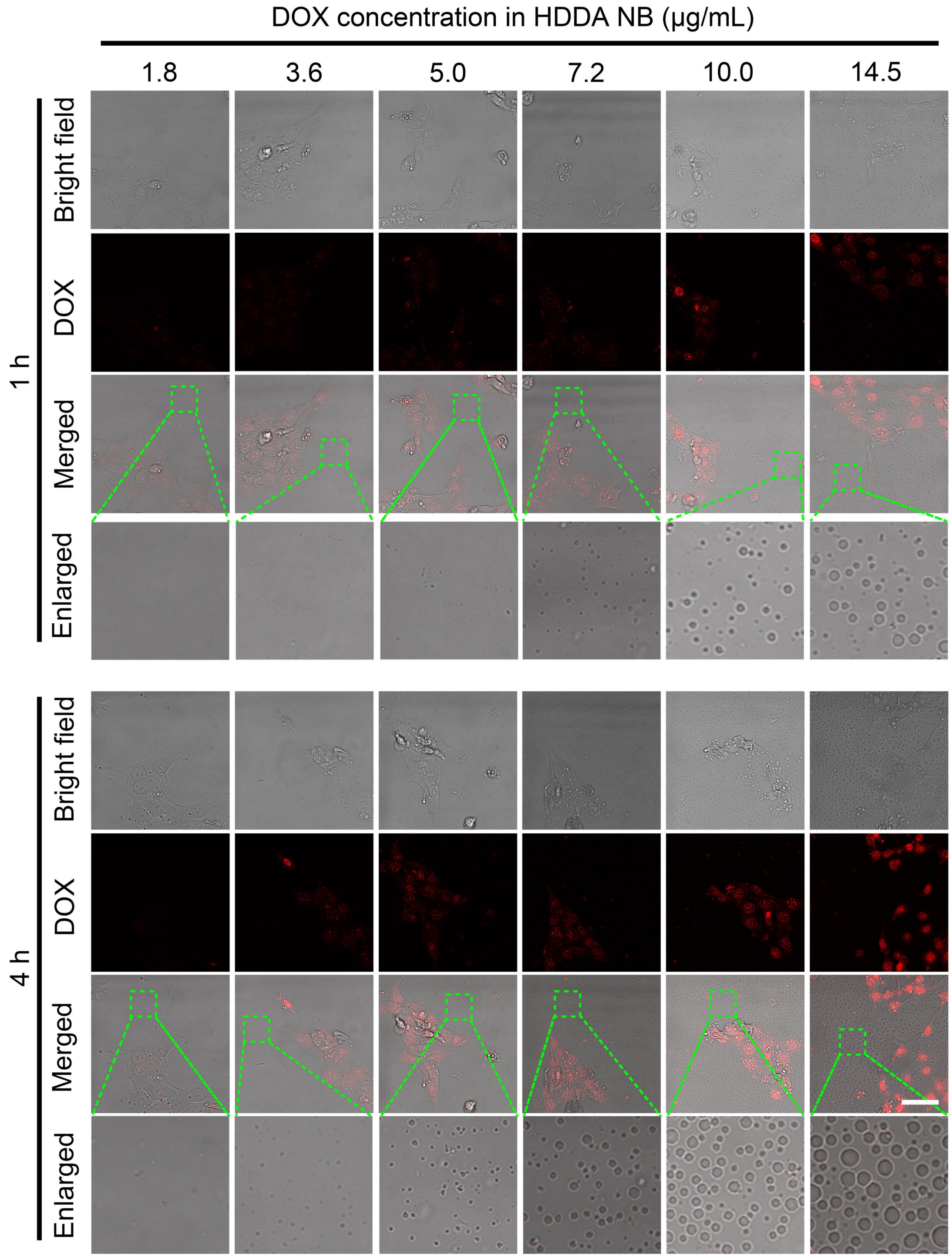

Supplementary Figure 9. Confocal microscopic images of the 4T1 cells after incubation with different concentrations of HDDA NBs (DOX concentrations in the HDDA NBs were 1.8, 3.6, 5.0, 7.2, 10.0, or 14.5 μg/mL, respectively.) for 1 or 4 h. Scale bar: 50 μm. The experiment was performed twice with similar results.

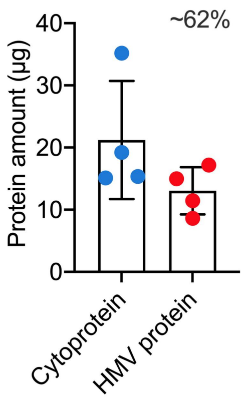

Supplementary Figure 10. Protein amounts of 1 × 10^5 4T1 cells (“Cytoprotein”) and HMVs produced by the same number of 4T1 cells (“HMV protein”). Data are presented as mean ± SD. n = 4 biologically independent samples per group.

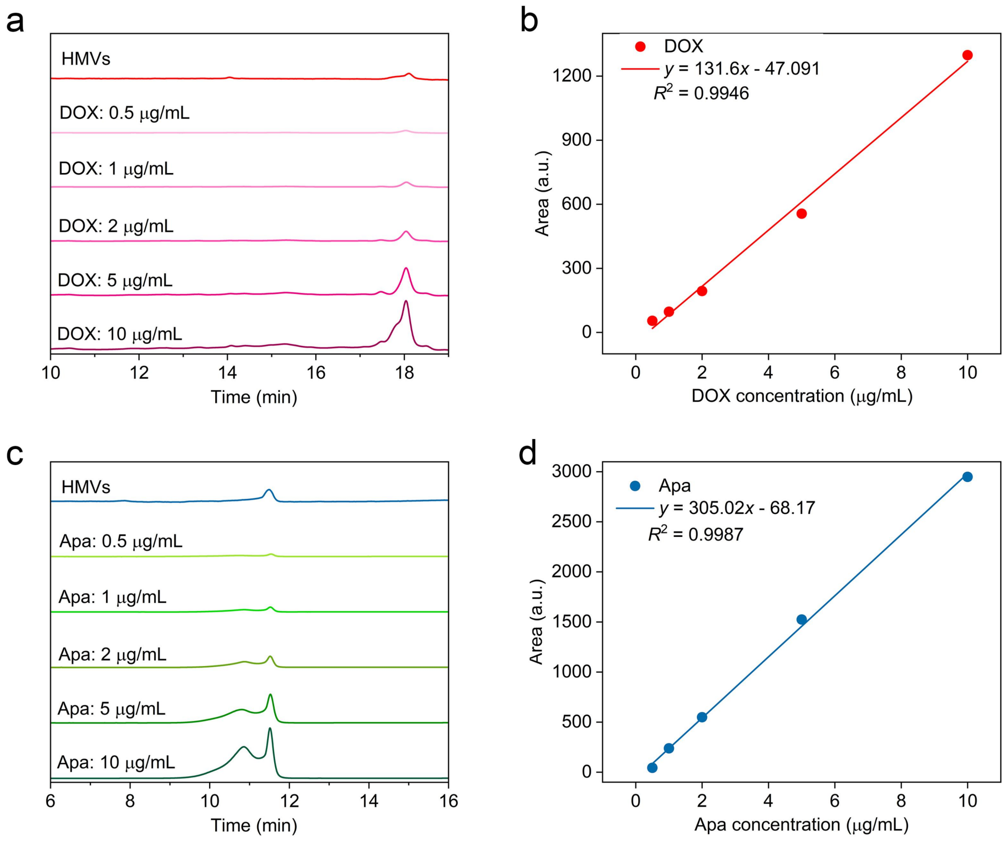

Supplementary Figure 11. (**a**) HPLC traces of HMVs and various concentrations of DOX. Wavelength for detection: 476 nm. (**b**) Linear fitting result of the area of the characteristic peak of DOX (between 17 min and 19 min) versus the DOX concentration. (**c**) HPLC traces of HMVs and various concentrations of Apa. Wavelength for detection: 341 nm. (**d**) Linear fitting result of the area of the characteristic peak of Apa (between 9 min and 12 min) versus

the Apa concentration. Protein concentration of the used HMVs: 5.8 mg/mL (determined by the BCA protein assay kit). All the samples were dispersed in 90% methanol/10% H_2O mixed solutions. The experiment was performed twice with similar results.

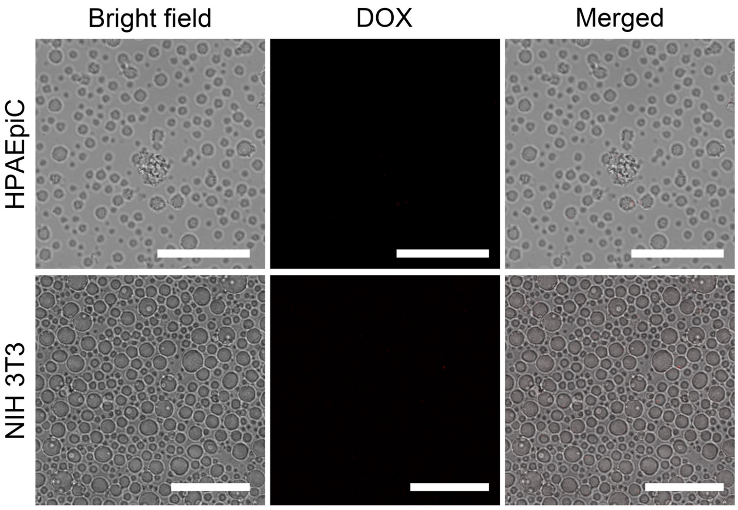

Supplementary Figure 12. Confocal microscopic images of HPAEpiC and NIH 3T3 cells after HDDA NB treatment. Scale bars: 30 μm. The DOX concentration in the HDDA NBs was 7.2 μg/mL. The experiment was performed three times with similar results.

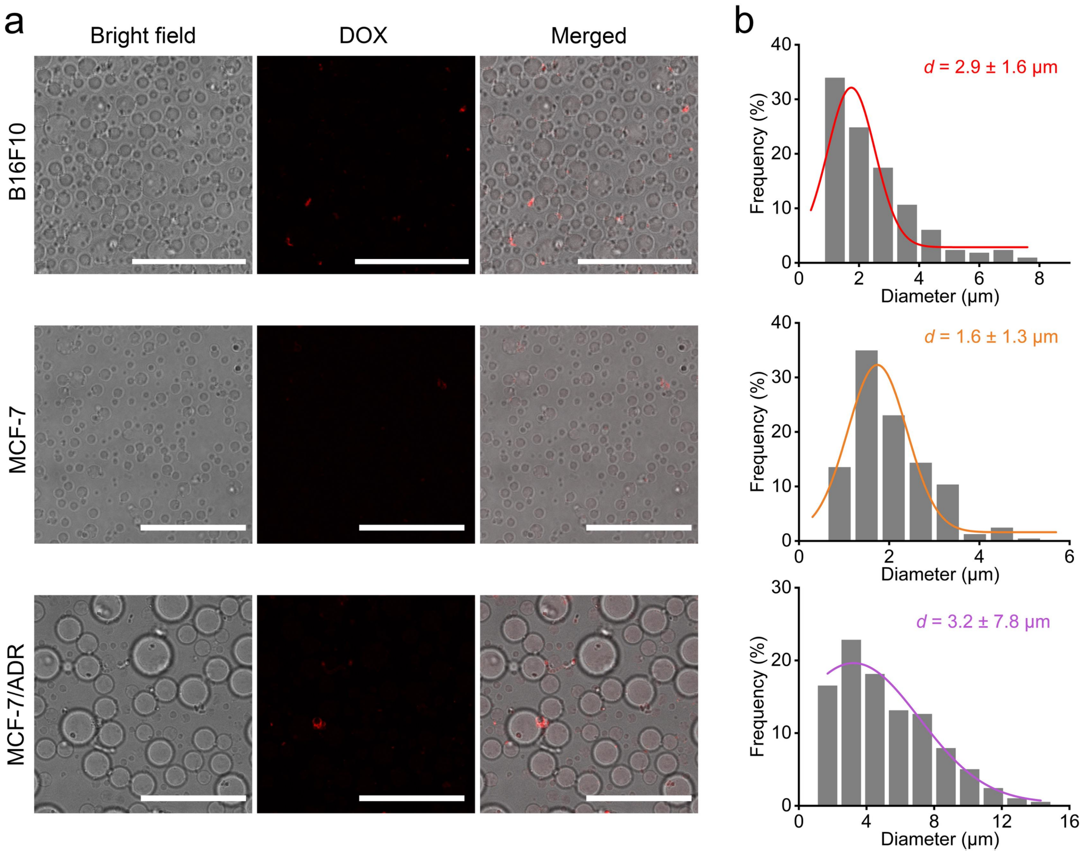

Supplementary Figure 13. (**a**) Confocal microscopic images and (**b**) corresponding statistic results of HDDA NBs-treated B16F10, MCF-7, and MCF-7/ADR cells. Scale bars: 30 μm. The DOX concentration in HDDA was 7.2 μg/mL. The experiment was performed twice with similar results.

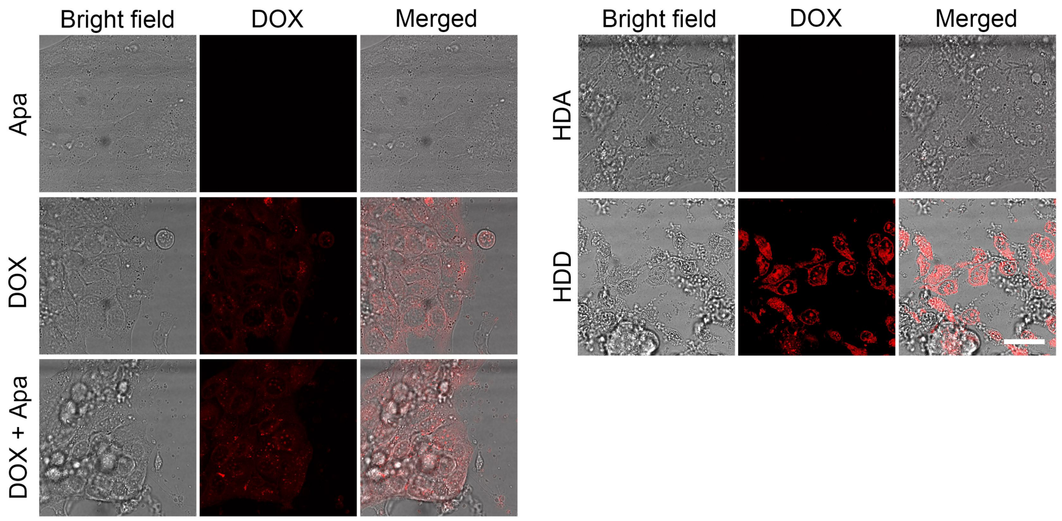

Supplementary Figure 14. Confocal microscopic images of 4T1 cells after Apa, DOX, DOX

+ Apa, HDA, or HDD treatment. Scale bar: 30 μm. The Apa and DOX concentrations in different Apa- or DOX-containing samples were 3.6 and 7.2 μg/mL, respectively. The experiment was performed three times with similar results.

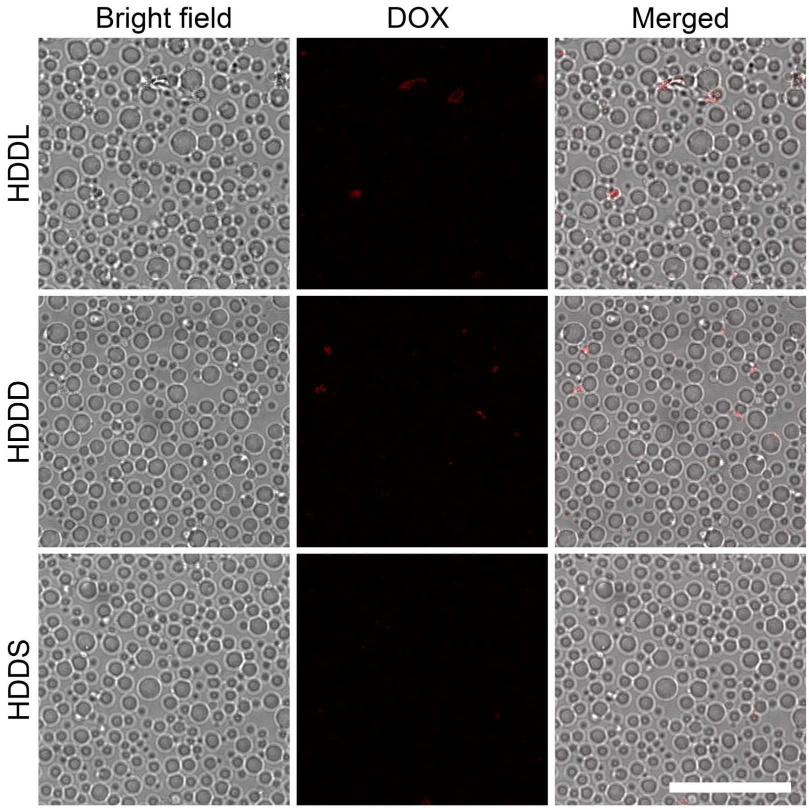

Supplementary Figure 15. Confocal microscopic images of 4T1 cells after HA−Den−DOX−Lap (HDDL), HA−Den−DOX−Das (HDDD), or HA−Den−DOX−Sor (HDDS) NB treatment. Scale bar: 30 μm. The DOX concentration in these samples was 7.2 μg/mL. The experiment was performed five times with similar results.

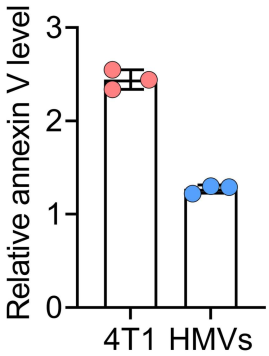

Supplementary Figure 16. Flow cytometric results showing the relative annexin V

expression levels in 4T1 cells and HMVs. Data are presented as mean ± SD. n = 3 biologically independent samples per group.

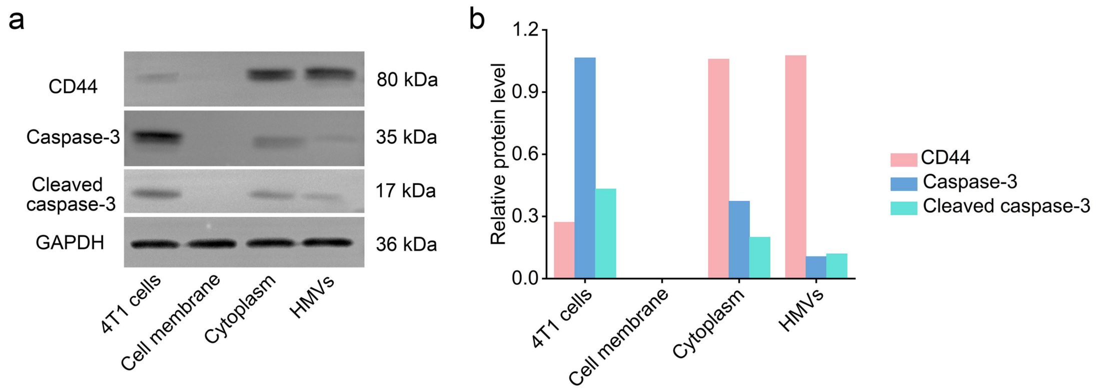

Supplementary Figure 17. (**a**) Western blot results and (**b**) corresponding statistical data of the CD44, caspase-3, and cleaved caspase-3 expression levels in the 4T1 cells, cell membranes (4T1), cytoplasm (4T1), and HMVs that were collected from 4T1 cell. GAPDH was used as the loading control. The experiment was performed twice with similar results.

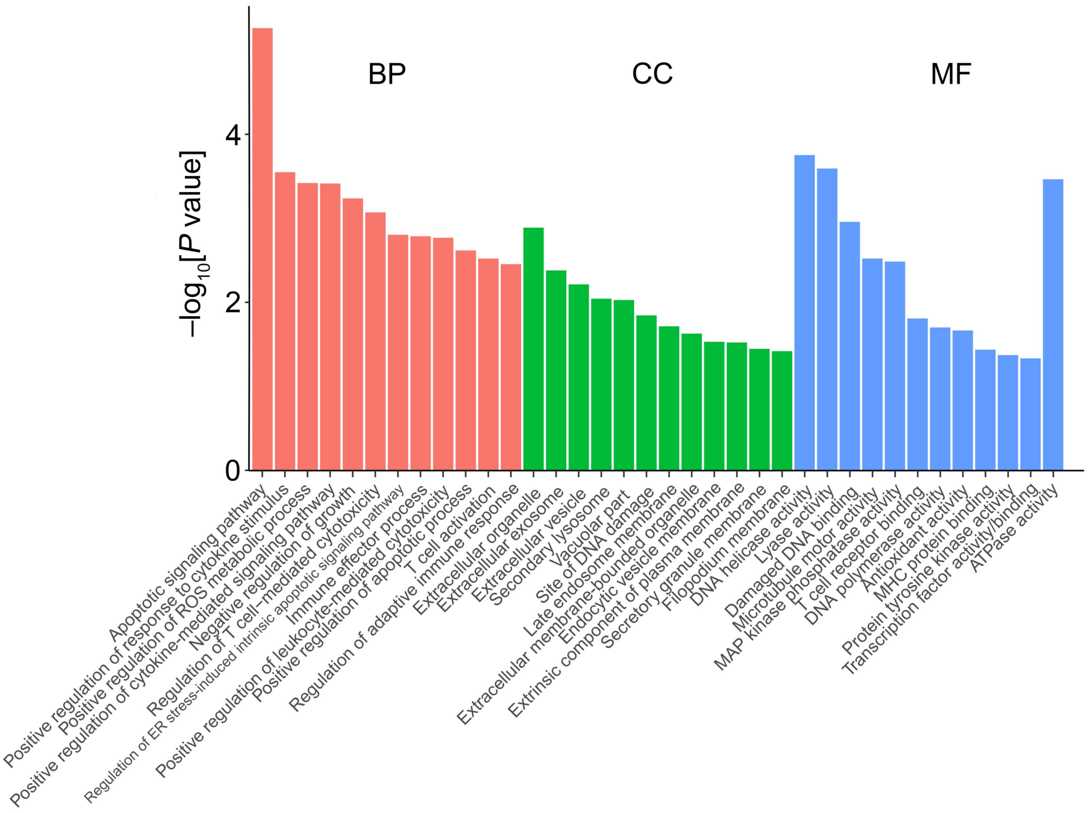

Supplementary Figure 18. Histogram showing the Gene Ontology (GO) enrichment analysis

results of selected differentially expressed genes (DEGs) between the culture medium (control)- and HDDA-treated 4T1 cells. ER: endoplasmic reticulum; MAP: mitogen-activated protein; MHC: major histocompatibility complex; ATPase: adenosine triphosphatase. BP: biological process; CC: cellular component; MF: molecular function. n = 2 biologically independent samples per group. Statistical significance was calculated via two-tailed Student's t-test.

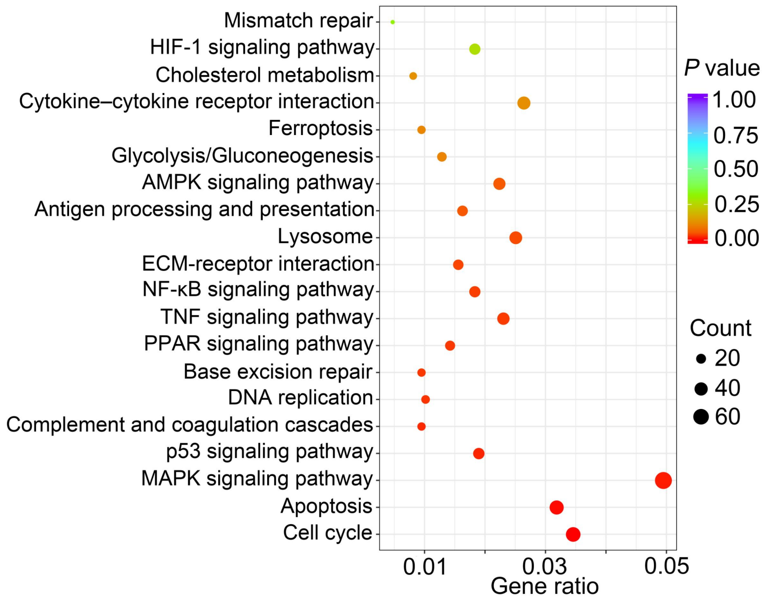

Supplementary Figure 19. Dot plot showing the Kyoto Encyclopedia of Genes and Genomes (KEGG) enrichment analysis results of selected DEGs between the culture medium (control)- and HDDA-treated 4T1 cells. HIF-1: hypoxia-inducible factor-1; AMPK: adenosine 5'-monophosphate-activated protein kinase; ECM: extracellular matrix; NF-κB: nuclear factor-κB; TNF: tumor necrosis factor; PPAR: peroxisome proliferator-activated receptor; MAPK: mitogen-activated protein kinase. Statistical significance was calculated via two-tailed Student's t-test.

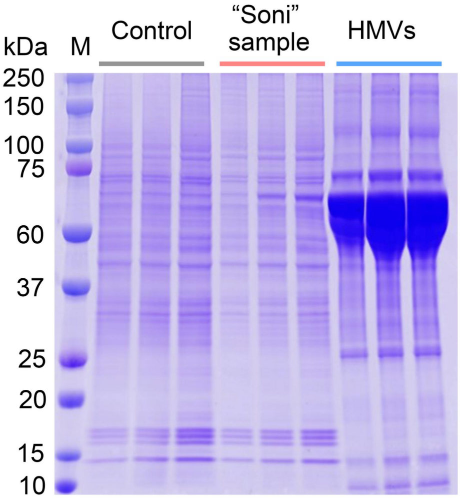

Supplementary Figure 20. SDS-PAGE results of the cytoproteins (control), "Soni" sample proteins, and HMV proteins in (or from) 4T1 cells. M: protein marker. The experiment was performed twice with similar results.

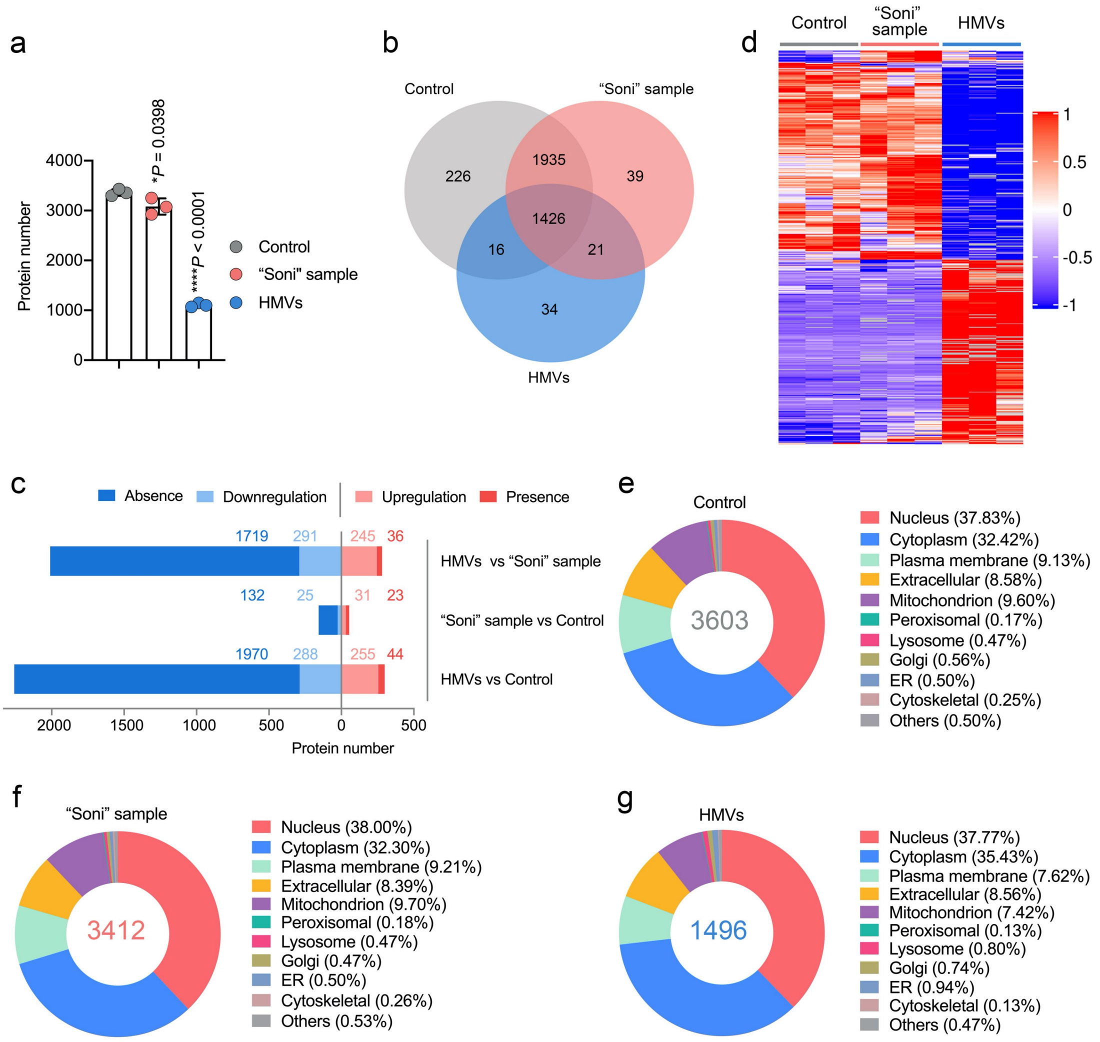

Supplementary Figure 21. Proteomic analyses of the cytoproteins (control), "Soni" sample proteins, and HMV proteins in (or from) 4T1 cells. (**a**) Statistics showing the detected number of protein types in each sample. Data are presented as mean ± SD. n = 3 biologically independent samples per group. *$P < 0.05$, ****$P < 0.0001$. (**b**) Venn diagram of the detected proteins in the control, "Soni" sample, and HMVs groups. The numbers in the figure indicate the number of the detected protein types. (**c**) Statistics showing the protein numbers of "presence", "absence", "upregulation", and "downregulation" for HMVs vs "Soni" sample, "Soni" sample vs Control, and HMVs vs Control, respectively. The numbers in the figure indicate the number of the protein types. (**d**) Heat map showing the levels of shared proteins in the control, "Soni" sample, and HMVs groups. Red: upregulation; blue: downregulation; P value < 0.05 and fold change > 2.0. (**e–g**) Cytolocalization of the detected proteins in the control, "Soni" sample, and HMVs groups, respectively. The numbers in the

middle of the cycles indicate the total number of the protein types. Statistical significance in (**a**) was calculated via one-way ANOVA with a Tukey's post-hoc test.

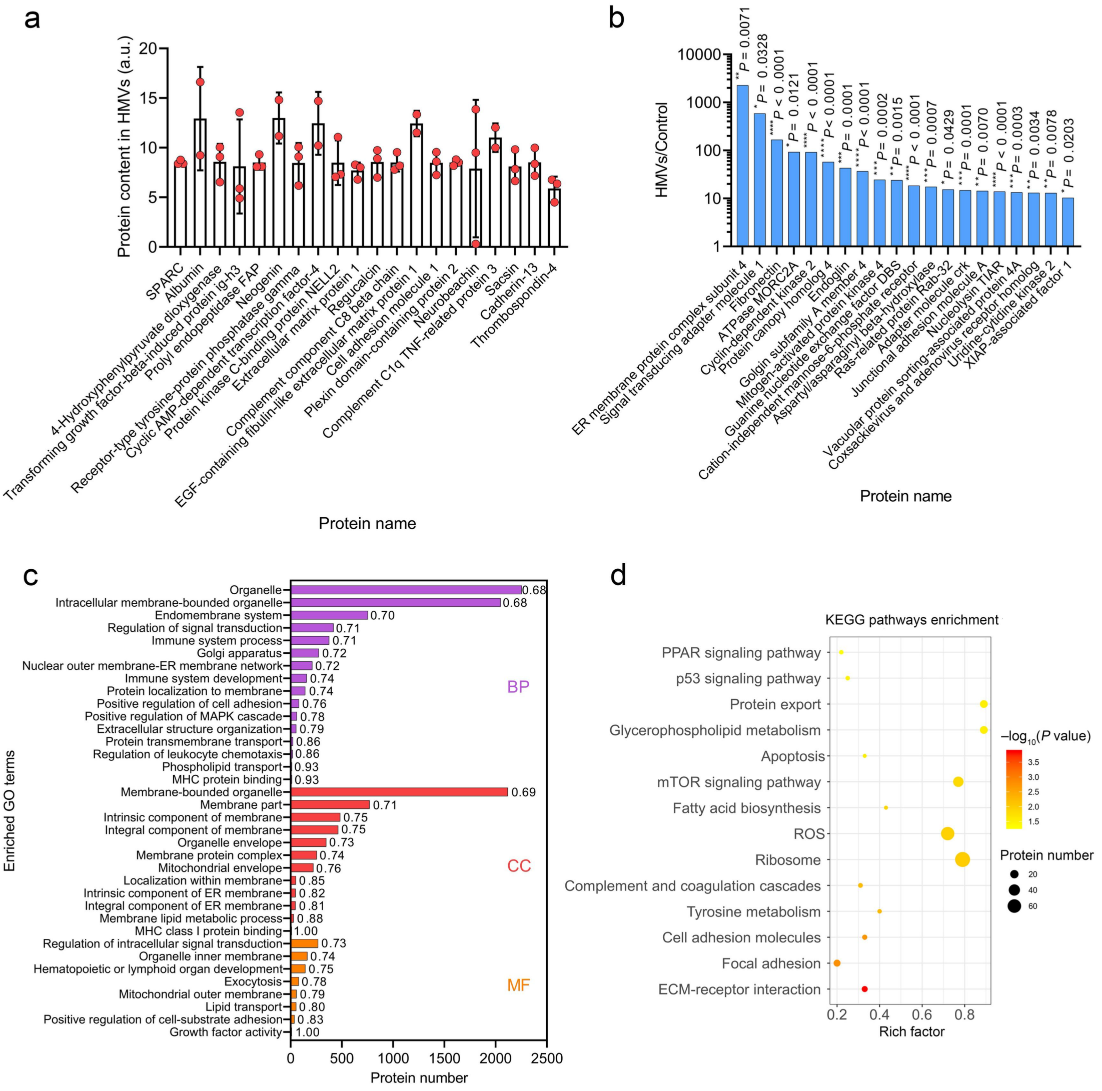

Supplementary Figure 22. Comparison of the proteomic results of the control and HMVs groups. (**a**) Representative proteins that are only detected in the HMVs group. Data are presented as mean ± SD. n = 3 biologically independent samples per group. SPARC: secreted protein, acidic and rich in cysteine, FAP: fibroblast activation protein, AMP: adenosine monophosphate, NELL2: neural epidermal growth factor-like 2, EGF: epidermal growth factor, TNF: tumor necrosis factor. (**b**) Representative proteins that are overexpressed in the HMVs group. Multiple of change > 10.0. n = 3 biologically independent samples per group. ER: endoplasmic reticulum, ATPase: adenosine triphosphatase, MORC2A: microrchidia 2A, TIAR: TIA-1 (T cell cytoplasmic antigen)-related nucleolysin, XIAP:

X-linked inhibitor of apoptosis protein. P value < 0.05 and fold change > 10.0. *P < 0.05, **P < 0.01, ***P < 0.001, ****P < 0.0001. (**c**) GO enrichment analysis results of the representative differentially expressed proteins (DEPs) that are overexpressed in the HMVs group. BP: biological process, MF: blue: molecular function, CC: cellular component, ER: endoplasmic reticulum, MAPK: mitogen-activated protein kinase, MHC: major histocompatibility complex. P value < 0.05. The numbers in the figure indicate the rich factors. (**d**) Dot plot showing the KEGG enrichment analysis results of representative DEPs between the HMVs group and the control group. P value < 0.05. PPAR: peroxisome proliferators-activated receptor, mTOR: mammalian target of rapamycin, ROS: reactive oxygen species, ECM: extracellular matrix. Statistical significance in (**b**,**d**) was calculated via two-tailed Student's t-test.

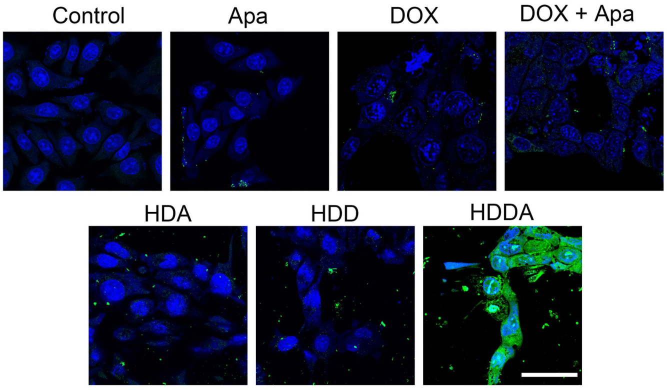

Supplementary Figure 23. Immunofluorescence images of 4T1 cells after different treatments for 24 h showing CRT protein expression (green, stained by anti-CRT primary antibody and FITC-labeled secondary antibody). Before imaging, the cell nuclei were visualized by Hoechst (blue). The DOX concentration in all the DOX-containing samples was 3.6 μg/mL. Scale bar: 40 μm. The experiment was performed three times with similar results.

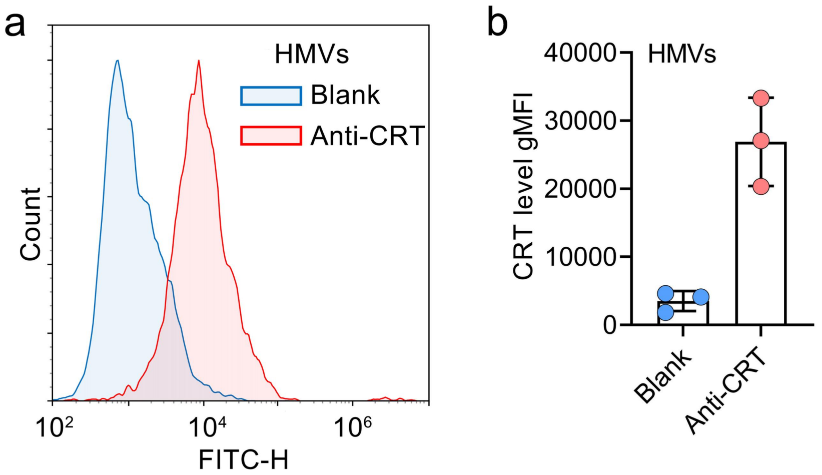

Supplementary Figure 24. Representative flow cytometric plots (**a**) and quantification results (**b**) of CRT protein expression (stained by anti-CRT primary antibody and FITC-labeled secondary antibody) level on the HMVs. The HMVs without antibody staining were set as the blank group. Data are presented as mean ± SD. $n = 3$ biologically independent samples per group.

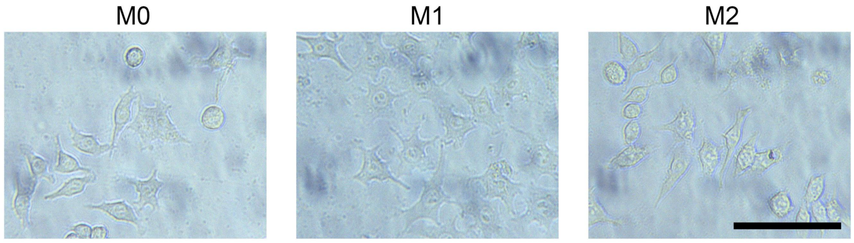

Supplementary Figure 25. The morphology of M0, M1, and M2 phenotypes of macrophages. Scale bar: 100 μm. The experiment was performed three times with similar results.

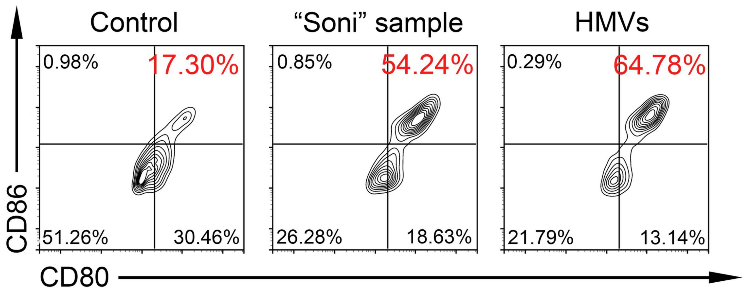

Supplementary Figure 26. Representative flow cytometric plots of matured DCs ($CD11c^+CD80^+CD86^+$) after incubation with culture medium (control), "Soni" sample, and HMVs, respectively. The experiment was performed twice with similar results.

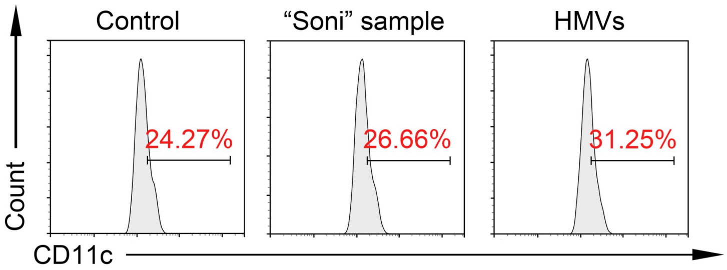

Supplementary Figure 27. Representative flow cytometric plots of M1-like macrophages ($F4/80^{+}CD11c^{+}$) after incubation with culture medium (control), "Soni" sample, and HMVs, respectively. The experiment was performed twice with similar results.

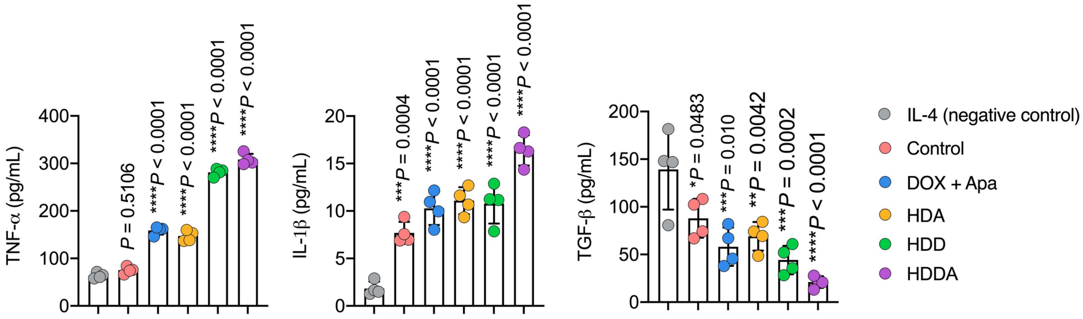

Supplementary Figure 28. Expression levels of TNF-α, IL-1β, and TGF-β produced by RAW 264.7 cells after different treatments, as analyzed by ELISA. The IL-4-incubated RAW 264.7 cells (M2-like macrophages) were set as the negative control. The culture media of the M2-like RAW 264.7 cells that were treated with the culture media of differently treated (as indicated in the figure) 4T1 cells were set as the control, DOX + Apa, HDA, HDD, and HDDA groups, respectively. Data are presented as mean ± SD. $n = 4$ biologically independent samples per group. $*P < 0.05$, $**P < 0.01$, $***P < 0.001$, $****P < 0.0001$. Statistical significance was calculated via one-way ANOVA with a Tukey's post-hoc test.

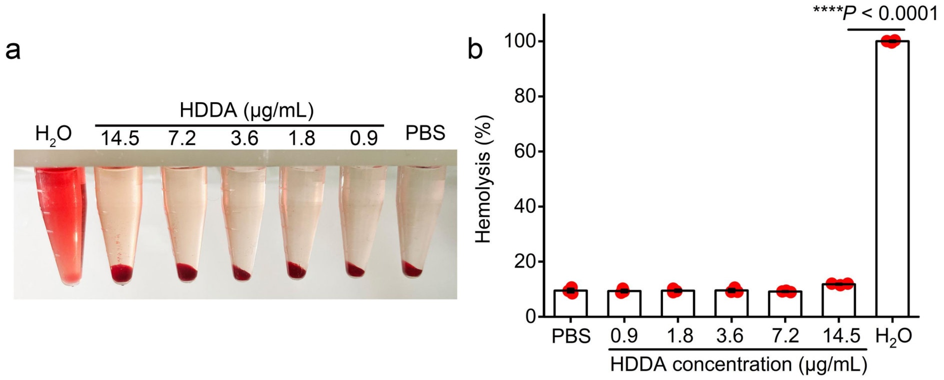

Supplementary Figure 29. (**a**) Photograph of red blood cell (RBC) suspensions after treatment with various concentrations (based on DOX) of HDDA NBs. The RBC suspensions treated with PBS and H_2O were set as negative control and positive control, respectively. (**b**) Corresponding hemolysis rates of the RBC samples in (**a**). Data are presented as mean ± SD. $n = 3$ biologically independent samples per group. ****$P < 0.0001$. Statistical significance in (**b**) was calculated via one-way ANOVA with a Tukey's post-hoc test.

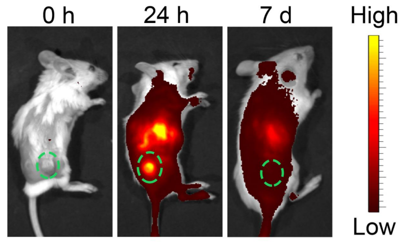

Supplementary Figure 30. Representative in vivo fluorescence images of 4T1 tumor-bearing BALB/c mice at different time points after the intravenous injection of DiR@HDDA NBs. The tumor regions of the mice were marked by green dotted circles. The experiment was performed three times with similar results.

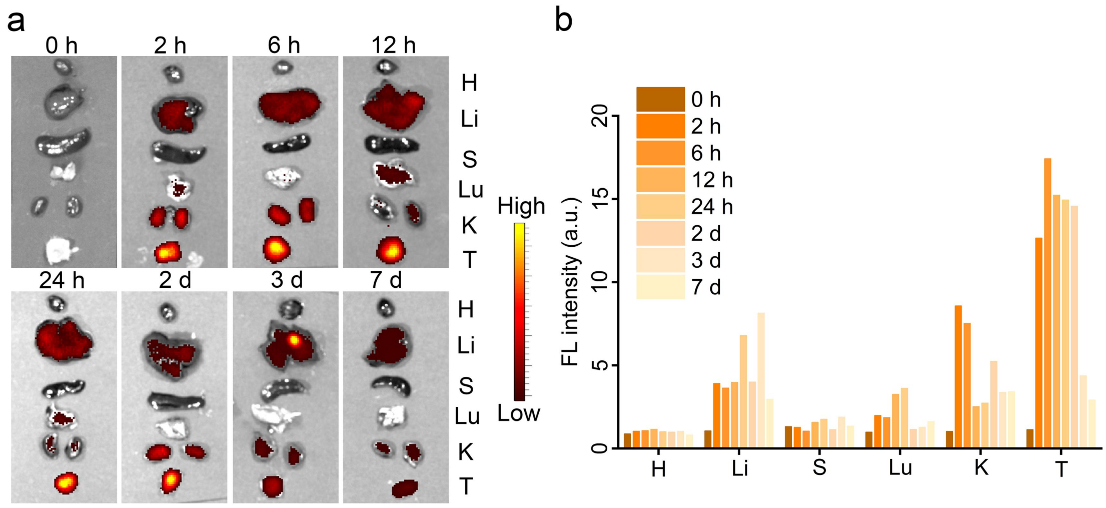

Supplementary Figure 31. (**a**) Representative ex vivo fluorescence images of major organs and tumor tissues excised from the mice sacrificed at different time points after the intravenous injection of DiR@HDDA and (**b**) corresponding fluorescence intensity results. H: heart, Li: liver, S: spleen, Lu: lung, K: kidneys, and T: tumor. The experiment was performed three times with similar results.

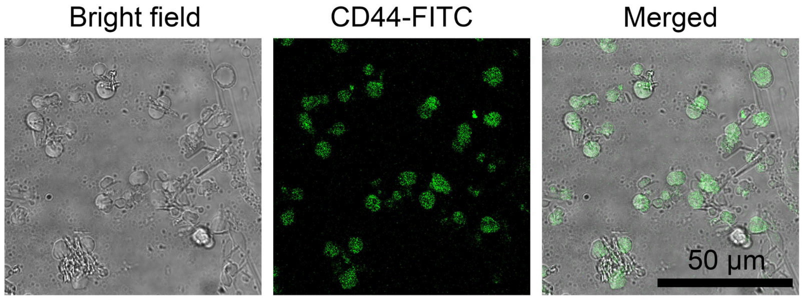

Supplementary Figure 32. Confocal microscopic images of the representative immunofluorescence staining results of the CD44 (green) proteins in HMVs collected from the HDDA-treated 4T1 cells. The experiment was performed twice with similar results.

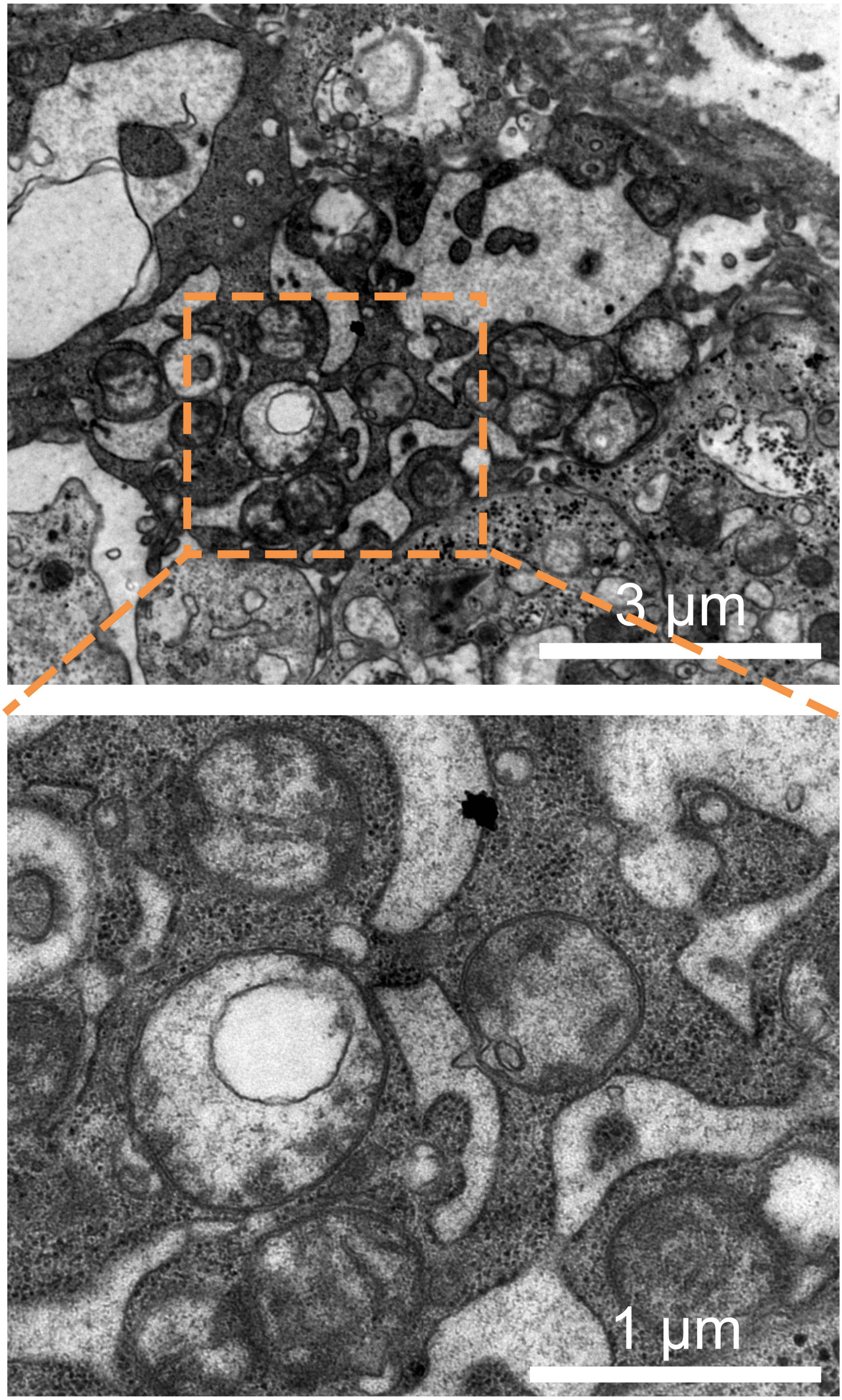
3 μm
1 μm

Supplementary Figure 33. Representative TEM images of the tumor tissue from a 4T1 tumor-bearing BALB/c mouse at day 3 after HDDA treatment. This figure is an enlarged version of Fig. 5i (without the blue dashed circles as marked in Fig. 5i). The experiment was performed twice with similar results.

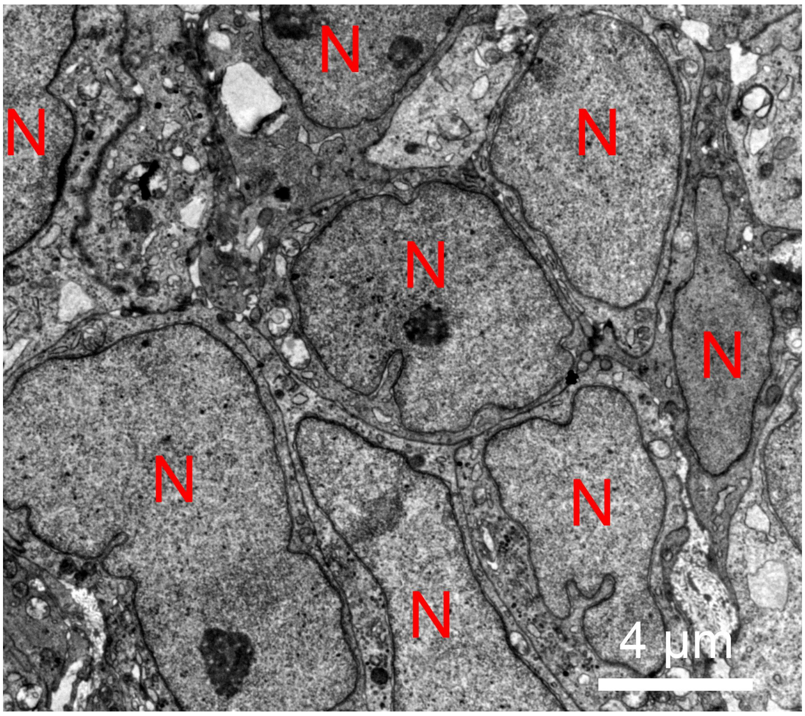

Supplementary Figure 34. Representative TEM image of the tumor tissue from a 4T1 tumor-bearing BALB/c mouse at day 3 after PBS treatment. N stands for nucleus. The experiment was performed twice with similar results.

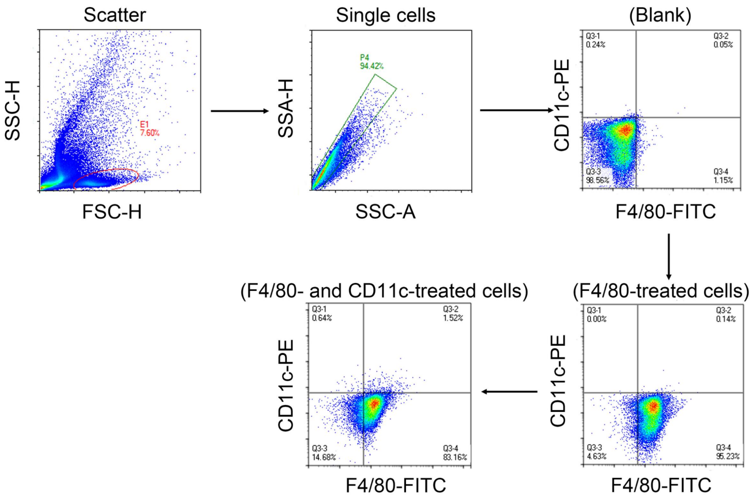

Supplementary Figure 35. Representative flow cytometry gating strategy for F4/80$^+$CD11c$^+$ M1-like TAMs. The experiment was performed twice with similar results.

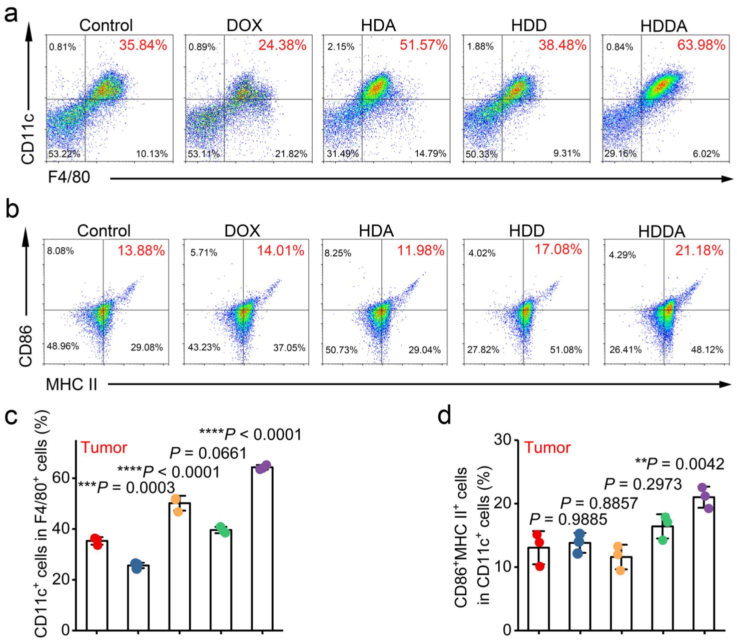

Supplementary Figure 36. Representative flow cytometric plots (**a**,**b**) and quantification results (**c**,**d**) of M1-like TAMs (F4/80$^+$CD11c$^+$) and matured DCs (CD11c$^+$CD86$^+$MHC II$^+$) in the 4T1 tumors retrieved from 4T1-bearing BALB/c mice 7 d after different treatments as indicated. Data are presented as mean ± SD. $n = 3$ mice per group. $^{**}P < 0.01$, $^{***}P < 0.001$, $^{****}P < 0.0001$. Statistical significance in (**c**,**d**) was calculated via one-way ANOVA with a Tukey's post-hoc test.

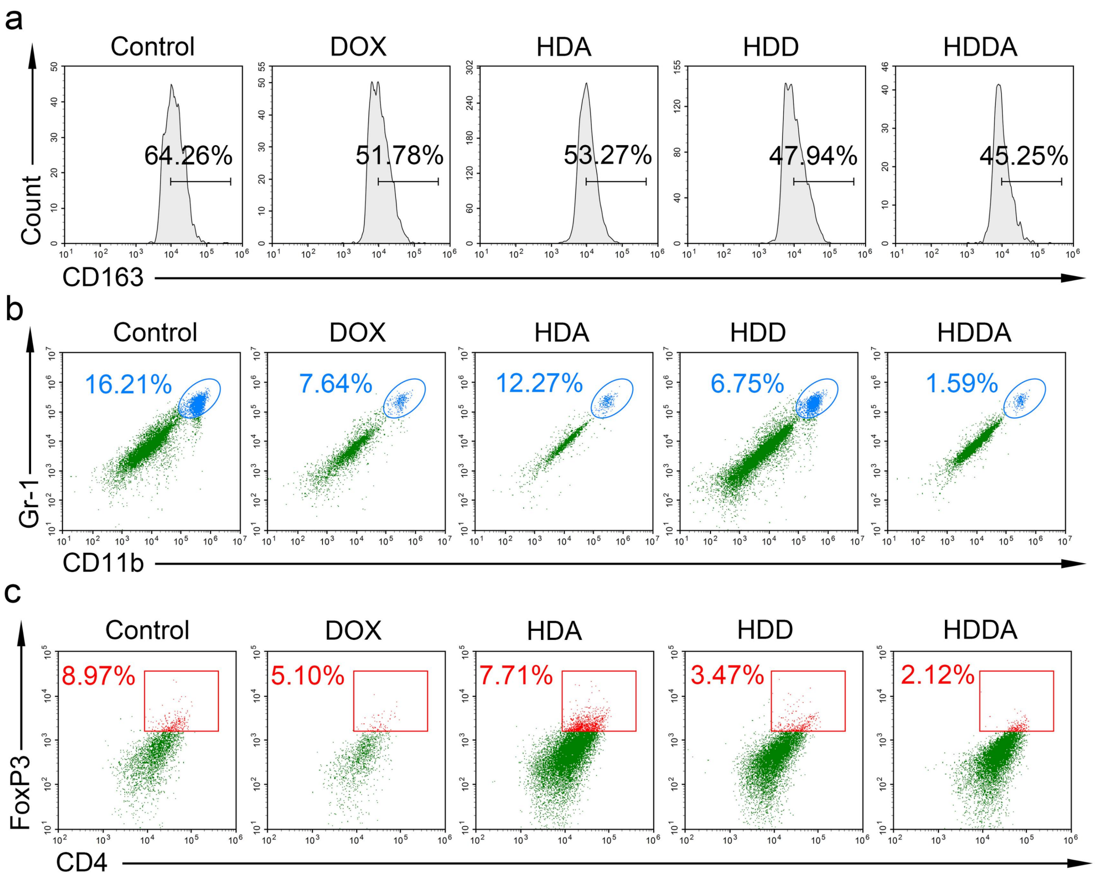

Supplementary Figure 37. Representative flow cytometric plots of M2-like macrophages (F4/80$^+$CD163$^+$) (**a**), MDSCs (CD11b$^+$Gr-1$^+$) (**b**), and Tregs (CD3$^+$CD4$^+$FoxP3$^+$) (**c**) in the tumor regions retrieved from the 4T1-bearing BALB/c mice 7 d after different treatments. The experiment was performed twice with similar results.

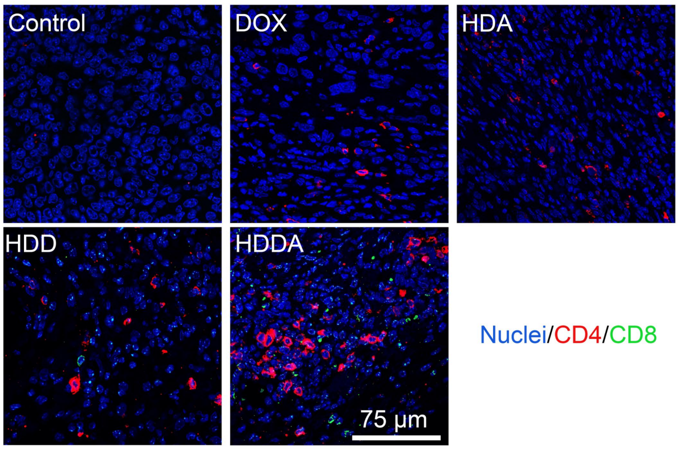

Supplementary Figure 38. Confocal fluorescence images of representative immunofluorescence staining results of CD4 (red) and CD8 (green) in tumor tissue slices

from the 4T1 tumor-bearing BALB/c mice sacrificed at day 7 after different treatments. CD4 and CD8 were stained with anti-CD4-FITC and anti-CD8-PE-Cy7 antibodies, respectively. Before imaging, the cell nuclei were stained with Hoechst. Scale bar: 75 μm. The experiment was performed twice with similar results.

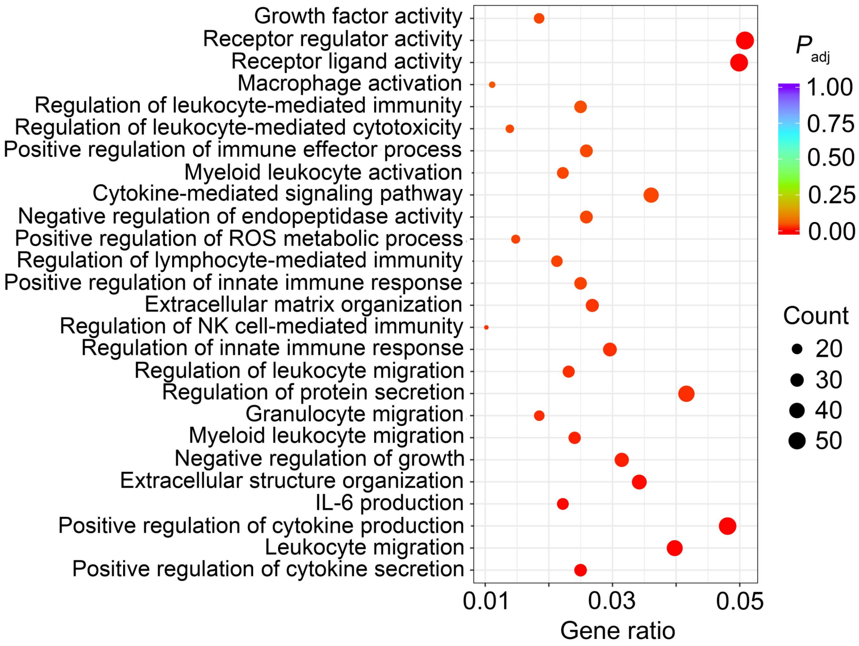

Supplementary Figure 39. Dot plot showing the GO enrichment analysis results of selected DEGs in 4T1 tumors from the mice after PBS (control) or HDDA treatment. P_{adj}: adjusted P value using Benjamini-Hochberg correction, NK: natural killer; IL-6: interleukin-6. n = 3 mice per group. Statistical significance was calculated via two-tailed Student's t-test.

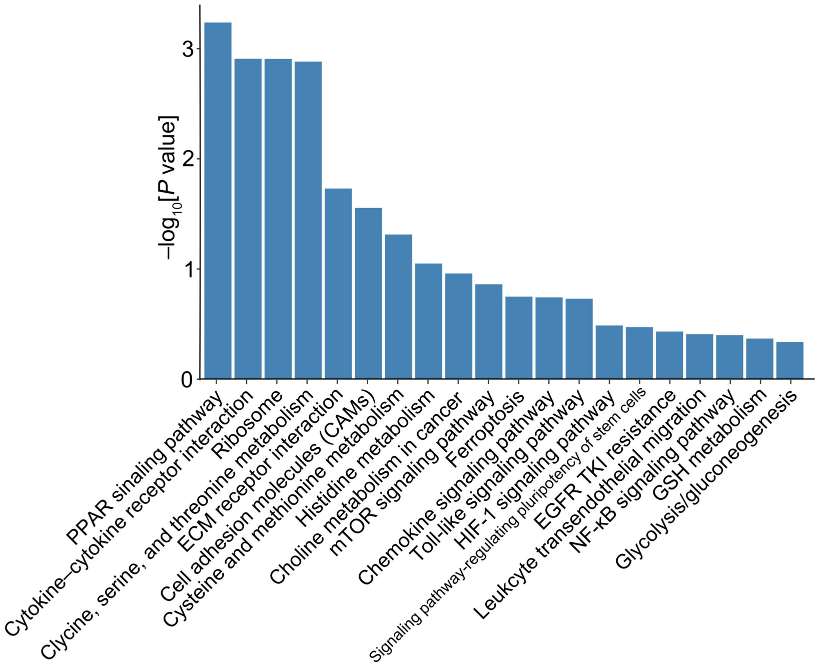

Supplementary Figure 40. Histogram showing the KEGG enrichment analysis results of selected DEGs in 4T1 tumors from the mice after PBS (control) or HDDA treatment. PPAR: peroxisome proliferator-activated receptor; ECM: extracellular matrix; mTOR: mammalian target of rapamycin; HIF-1: hypoxia-inducible factor-1; EGFR: epidermal growth factor receptor; TKI: tyrosine kinase inhibitor; NF-κB: nuclear factor-κB; GSH: glutathione. n = 3 mice per group. Statistical significance was calculated via two-tailed Student's t-test.

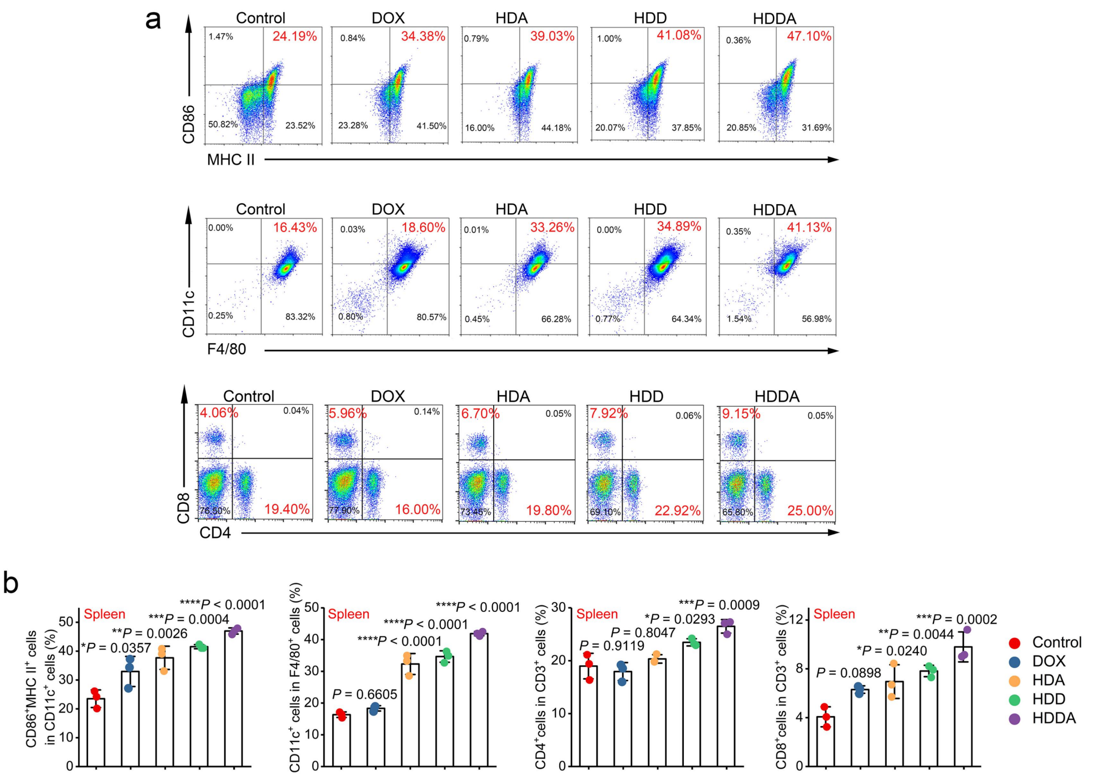

Supplementary Figure 41. Representative flow cytometric plots (**a**) and quantification results (**b**) of matured DCs ($CD11c^{+}CD86^{+}MHC\ II^{+}$), M1-like macrophages ($F4/80^{+}CD11c^{+}$), cytotoxic T cells ($CD3^{+}CD8^{+}$), and helper T cells ($CD3^{+}CD4^{+}$) in the spleens retrieved from 4T1-bearing BALB/c mice 7 d after different treatments as indicated. Data are presented as mean ± SD. n = 3 mice per group. *P < 0.05, **P < 0.01, ***P < 0.001, ****P < 0.0001. Statistical significance in (**b**) was calculated via one-way ANOVA with a Tukey's post-hoc test.

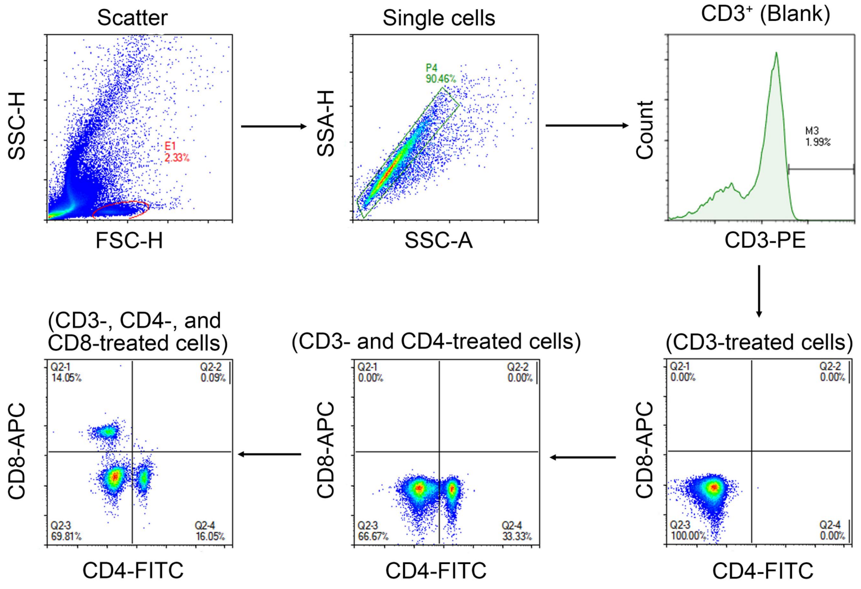

Supplementary Figure 42. Representative flow cytometry gating strategy for $CD3^+CD4^+$ or $CD3^+CD8^+$ T cells. The experiment was performed twice with similar results.

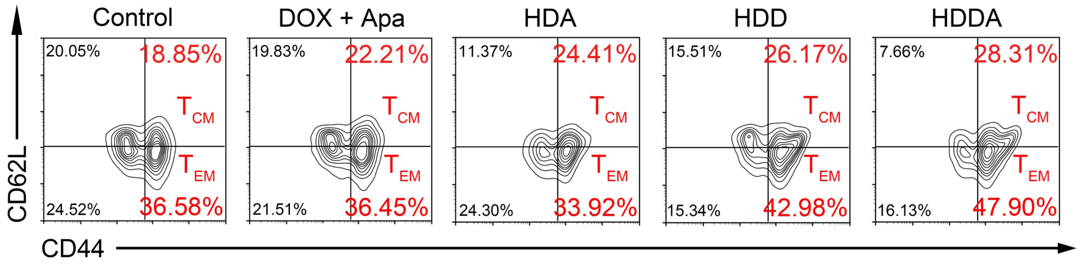

Supplementary Figure 43. Representative flow cytometric plots of T_{CM} ($CD3^+CD8^+CD44^+CD62L^+$) and T_{EM} ($CD3^+CD8^+CD44^+CD62L^-$) in the spleens retrieved from the 4T1-bearing BALB/c mice 30 d after different treatments.

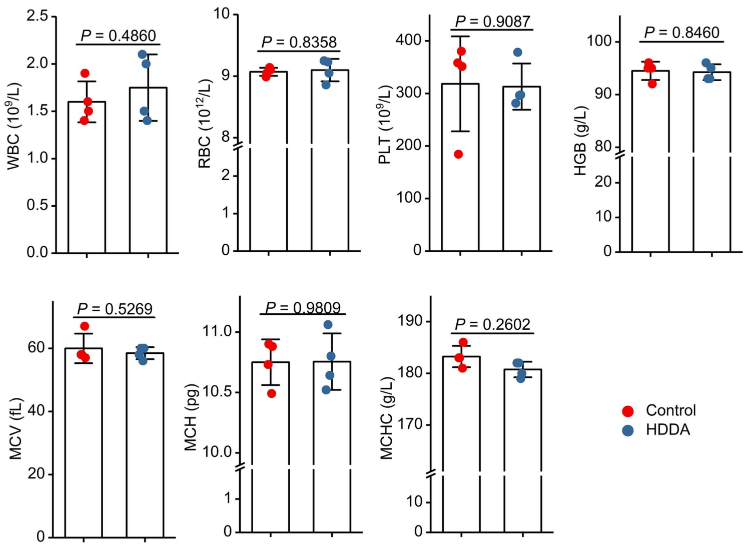

Supplementary Figure 44. Routine blood analysis results of the BALB/c mice collected on the 7th day after the intravenous injection of PBS (control) or HDDA suspension (DOX dose: 5 mg/kg; n = 4/group). Data are presented as mean ± SD. The blood indexes including WBC, RBC, PLT, HGB, MCV, MCH, and MCHC indicate the numbers of white blood cells, red blood cells, and platelets, concentration of hemoglobin, mean corpuscular volume, mean corpuscular hemoglobin, and mean corpuscular hemoglobin concentration, respectively. Statistical significance was calculated via two-tailed Student's t-test.

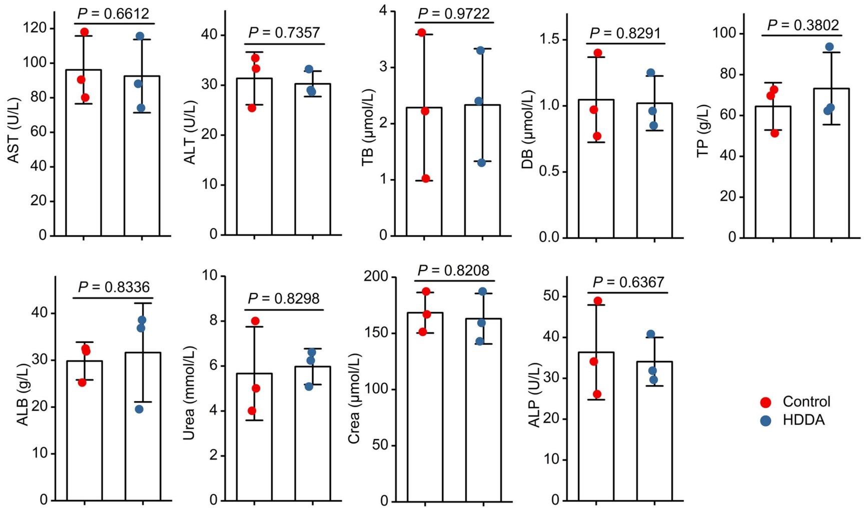

Supplementary Figure 45. Biochemical analysis results of the blood samples collected from the BALB/c mice sacrificed on the 7th day after treatment with PBS (control) or HDDA suspension (DOX dose: 5 mg/kg; n = 3/group). Data are presented as mean ± SD. The

corresponding indexes include aspartate transferase (AST), alanine transferase (ALT), total bilirubin (TB), direct bilirubin (DB), total protein (TP), albumin (ALB), urea, creatinine (Crea), and alkaline phosphatase (ALP). Statistical significance was calculated via two-tailed Student's *t*-test.

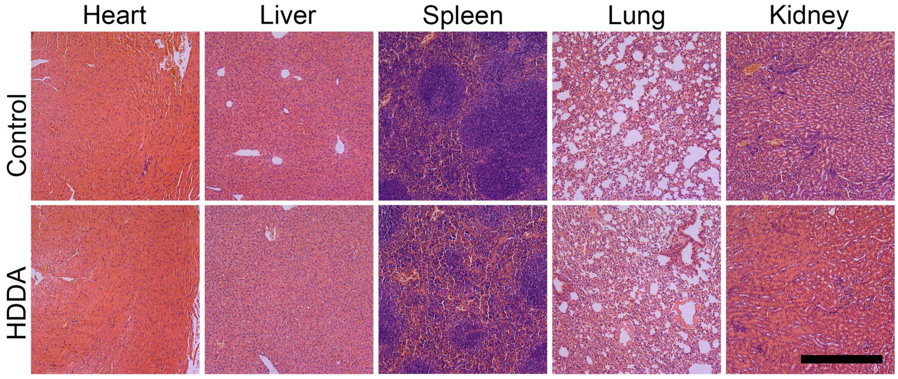

Supplementary Figure 46. H&E-stained tissue slices of the major organs (hearts, livers, spleens, lungs, and kidneys) excised from the BALB/c mice sacrificed on the 7th day after intravenous injection of PBS (control) or HDDA suspension (DOX dose: 5 mg/kg), respectively. Scale bar: 150 μm. $n = 3$ mice per group. The experiment was performed twice with similar results.

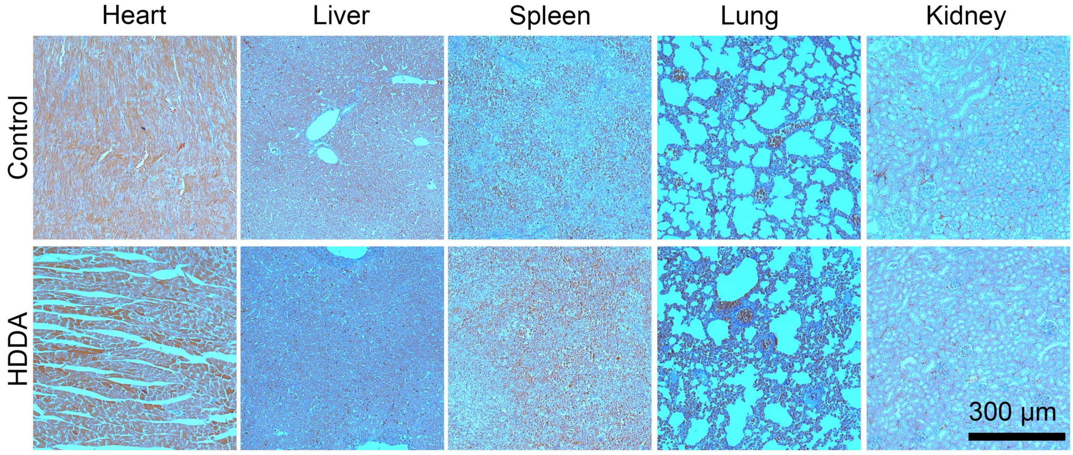

Supplementary Figure 47. Masson-stained tissue slices of the major organs (hearts, livers, spleens, lungs, and kidneys) excised from the BALB/c mice sacrificed on the 14th day after intravenous injection of PBS (control) or HDDA suspension (DOX dose: 5 mg/kg). $n = 3$ mice per group. The experiment was performed twice with similar results.

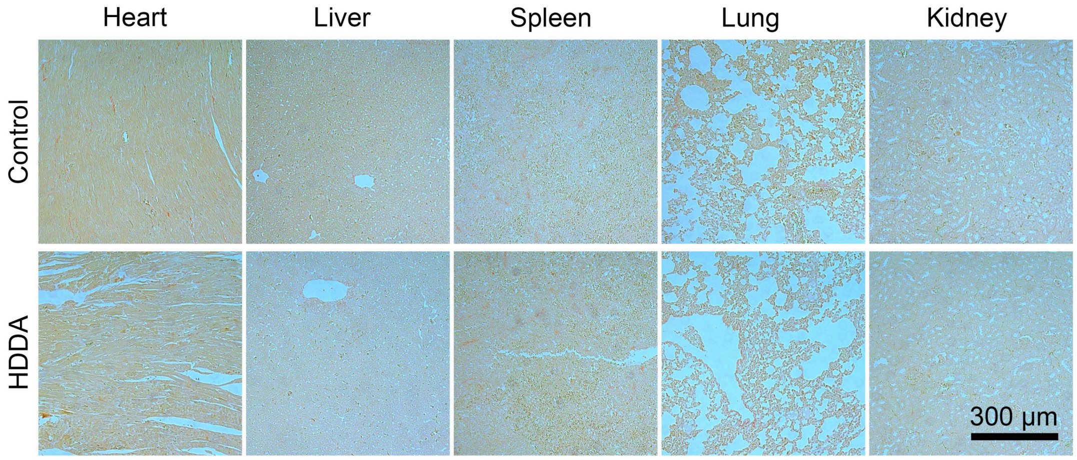

Supplementary Figure 48. Van Gieson (VG)-stained tissue slices of the major organs (hearts, livers, spleens, lungs, and kidneys) excised from the BALB/c mice sacrificed on the 14th day after intravenous injection of PBS (control) or HDDA suspension (DOX dose: 5 mg/kg). $n = 3$ mice per group. The experiment was performed twice with similar results.

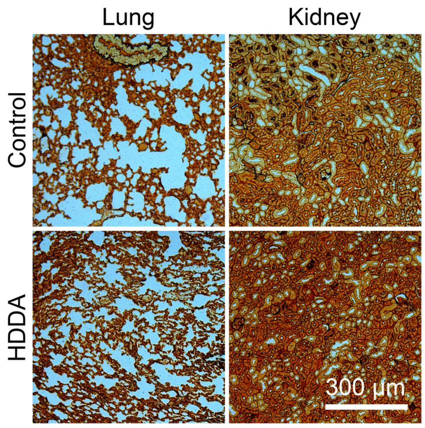

Supplementary Figure 49. Hexamine silver-stained tissue slices of the lungs and kidneys excised from the BALB/c mice sacrificed on the 14th day after intravenous injection of PBS (control) or HDDA suspension (DOX dose: 5 mg/kg). $n = 3$ mice per group. The experiment was performed twice with similar results.

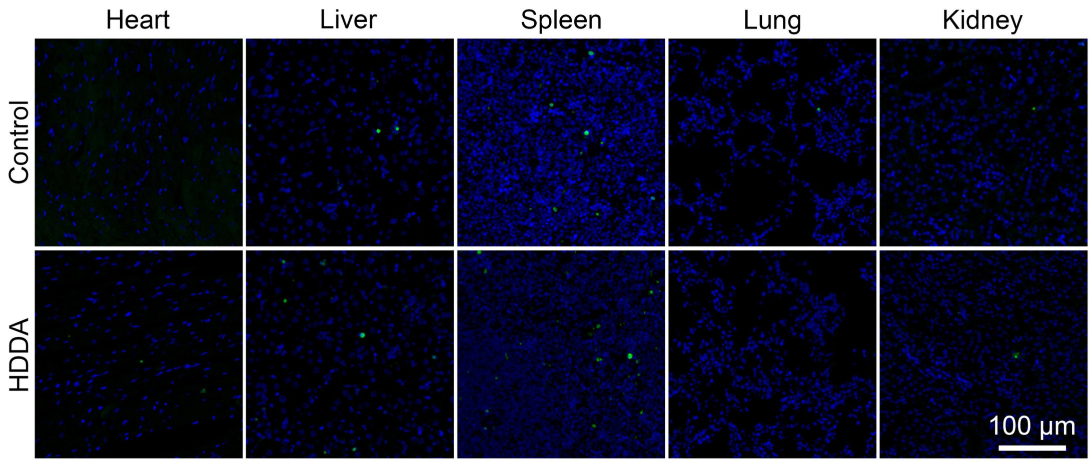

Supplementary Figure 50. Confocal microscopic images of representative TUNEL assay results of the major organs (hearts, livers, spleens, lungs, and kidneys) excised from the BALB/c mice sacrificed on the 14th day after intravenous injection of PBS (control) or HDDA suspension (DOX dose: 5 mg/kg). Before imaging, the cell nuclei were stained with Hoechst. $n = 3$ mice per group. The experiment was performed twice with similar results.

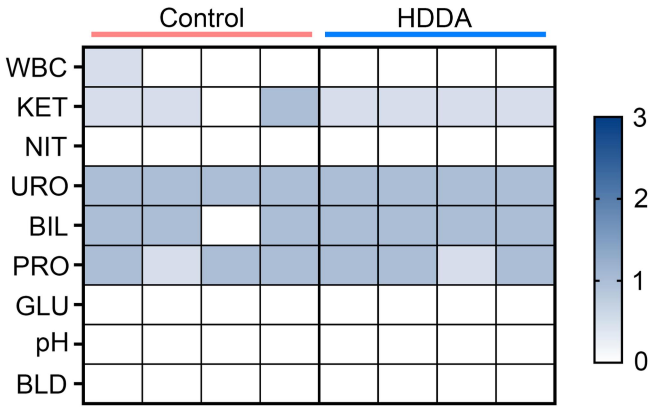

Supplementary Figure 51. Semiquantitative urine routine test results of the BALB/c mice collected on the 14th day after intravenous injection of PBS (control) or HDDA suspension (DOX dose: 5 mg/kg). $n = 4$/group. The urine indexes including WBC, KET, NIT, URO, BIL, PRO, GLU, pH, and BLD indicate the number of white blood cells, concentration of ketone body, nitrite, urobilinogen, bilirubin, protein, and glucose, the pH value, and the occult blood, respectively.

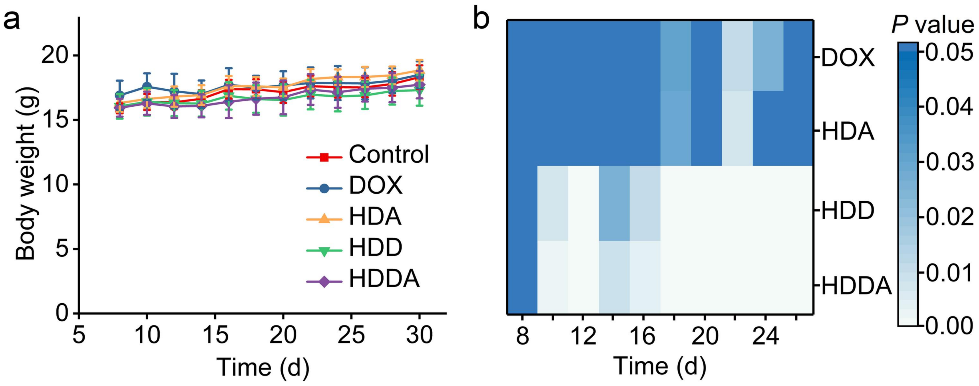

Supplementary Figure 52. (**a**) Body weight changes of the 4T1 tumor-bearing BALB/c mice after various treatments (n = 9), and (**b**) corresponding heat map of the P values between the PBS-treated group and other groups. The mice treated with PBS were set as the control group. Data are presented as mean ± SD. Statistical significance in (**b**) was calculated via one-way ANOVA with a Tukey's post-hoc test.

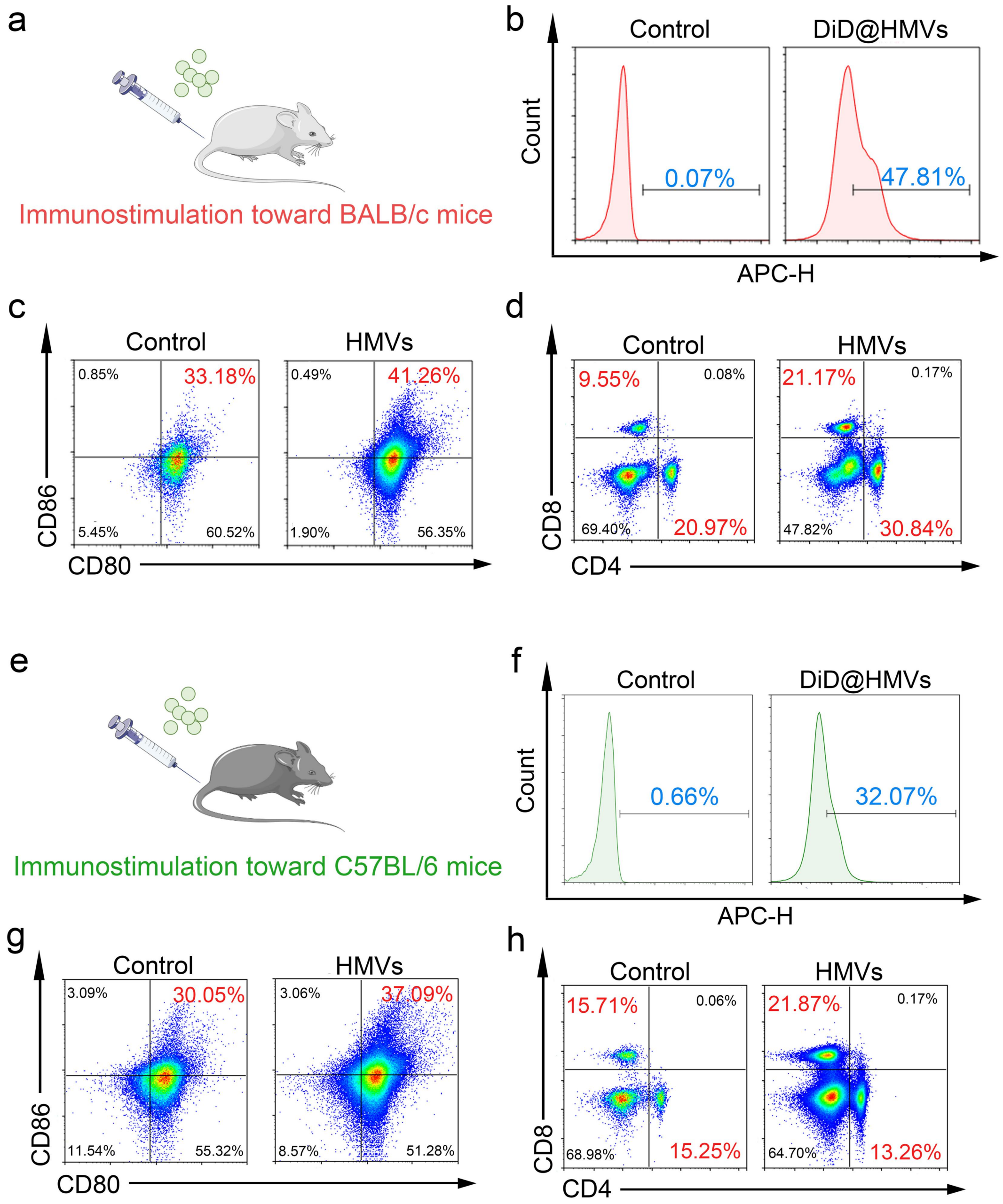

Supplementary Figure 53. Systemic anticancer immunostimulation induced by HMVs. (**a**) Scheme showing the subcutaneous treatment of healthy BALB/c mice with HMVs. (**b**) Representative flow cytometric plots of the cells retrieved from the lymph nodes of the BALB/c mice subcutaneously (s.c.) injected with PBS (control) or DiD@HMVs. (**c**,**d**) Representative flow cytometric plots of the matured DCs ($CD11c^{+}CD86^{+}CD80^{+}$) (**c**) and the cytotoxic T cells ($CD3^{+}CD8^{+}$) and helper T cells ($CD3^{+}CD4^{+}$) (**d**) retrieved from the lymph nodes of the BALB/c mice s.c. injected with PBS (control) or HMVs. (**e**) Scheme showing the subcutaneous treatment of healthy C57BL/6 mice with HMVs. (**f**) Representative flow

cytometric plots of the cells retrieved from the lymph nodes of the C57BL/6 mice s.c. injected with PBS (control) or DiD@HMVs. (**g**,**h**) Representative flow cytometric plots of the matured DCs ($CD11c^+CD86^+CD80^+$) (**g**) and the cytotoxic T cells ($CD3^+CD8^+$) and helper T cells ($CD3^+CD4^+$) (**h**) retrieved from the lymph nodes of the C57BL/6 mice s.c. injected with PBS (control) or HMVs.

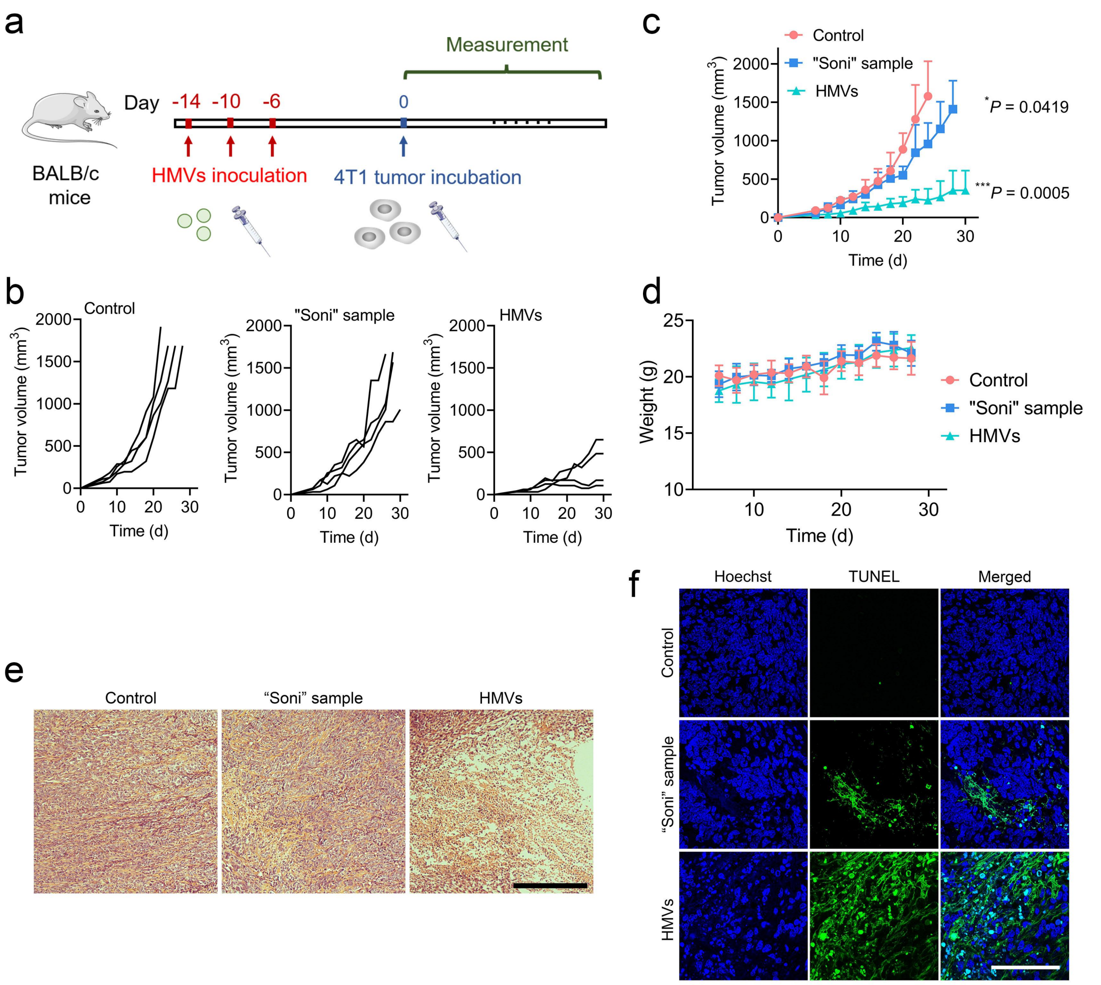

Supplementary Figure 54. HMV as cancer vaccines for tumor prevention. (**a**) Experimental procedure for assessing the 4T1 tumor prevention capacity of the "Soni" sample or HMVs in BALB/c mice. (**b**,**c**) Individual 4T1 tumor growth curves (**b**) and average 4T1 tumor growth curves (**c**) of the BALB/c mice pretreated with PBS (control), "Soni" sample, or HMVs. Data are presented as mean + SD. (**d**) Body weight changes of the 4T1-bearing BALB/c mice after different treatments (n = 4/group). Data are presented as mean ± SD. (**e**,**f**) Confocal microscopic images of representative H&E staining results (scale bar: 300 μm) (**e**)

and TUNEL assay results (scale bar: 100 μm) (**f**) of the tumor tissue slices from the BALB/c mice pretreated with PBS (control), "Soni" sample, or HMVs before 4T1 tumor implantation. For (**f**), before imaging, the cell nuclei were stained with Hoechst. For (**e**,**f**), n = 3 mice per group and the experiments were performed twice with similar results. Statistical significance in (**c**) was calculated via one-way ANOVA with a Tukey's post-hoc test.

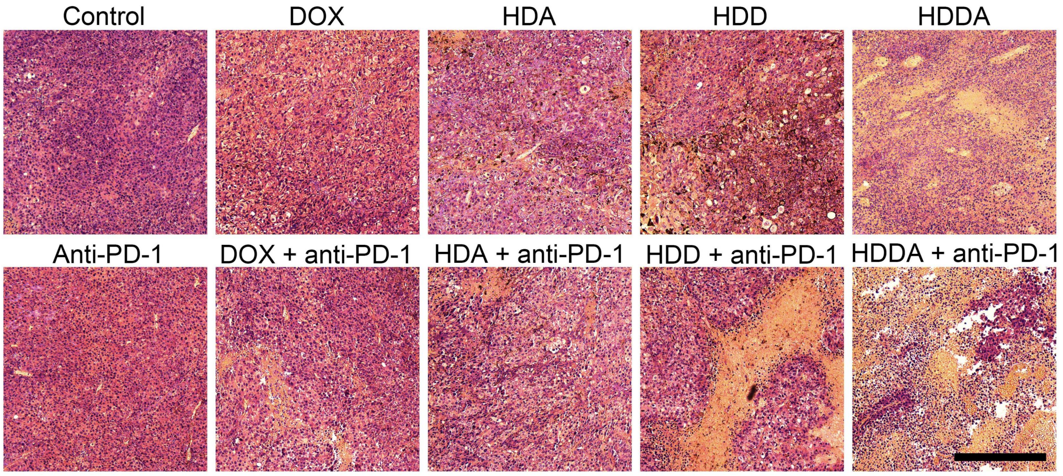

Supplementary Figure 55. Microscopic images of the H&E-stained tumor tissues of the B16F10 tumor-bearing C57BL/6 mice taken at day 7 after various treatments as indicated. Scale bar: 300 μm. n = 3 mice per group. The experiment was performed twice with similar results.

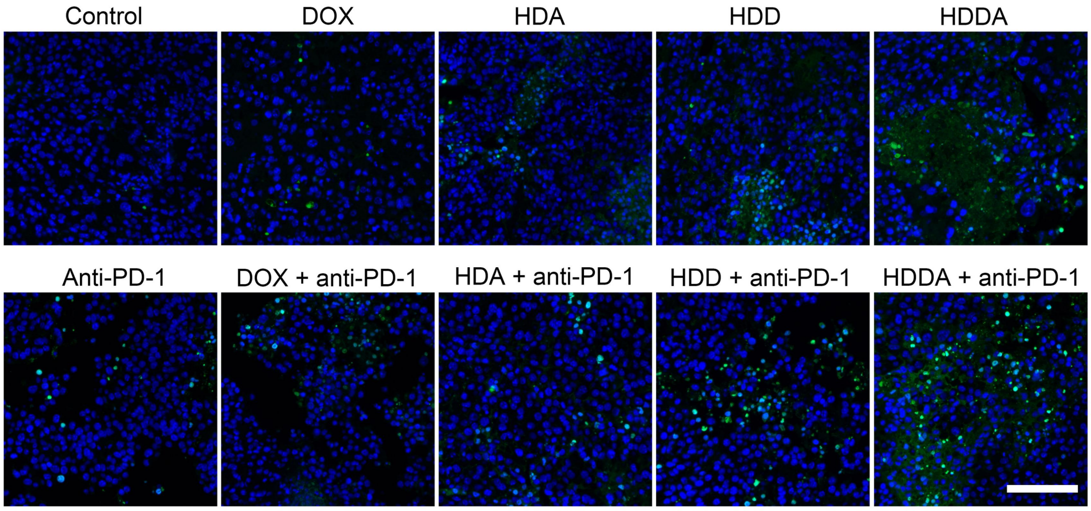

Supplementary Figure 56. Confocal fluorescence images of the representative TUNEL assay

results of the tumor tissue slices from the B16F10 tumor-bearing C57BL/6 mice sacrificed at day 7 after different treatments. Before imaging, the cell nuclei were stained with Hoechst. Scale bar: 100 μm. n = 3 mice per group. The experiment was performed twice with similar results.

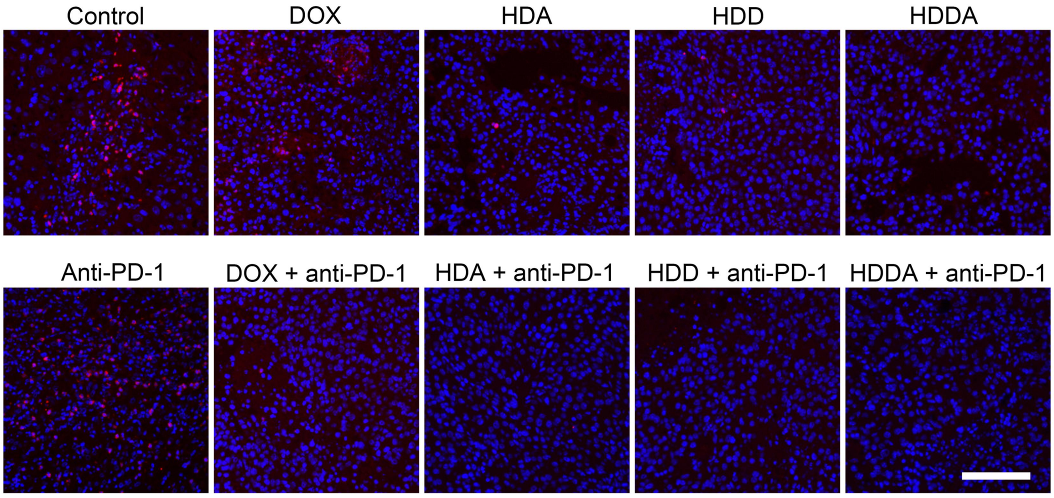

Supplementary Figure 57. Confocal fluorescence images of the representative immunofluorescence staining results of Gr-1 in tumor tissue slices from the B16F10 tumor-bearing C57BL/6 mice sacrificed at day 7 after different treatments. Scale bar: 100 μm. n = 3 mice per group. The experiment was performed twice with similar results.

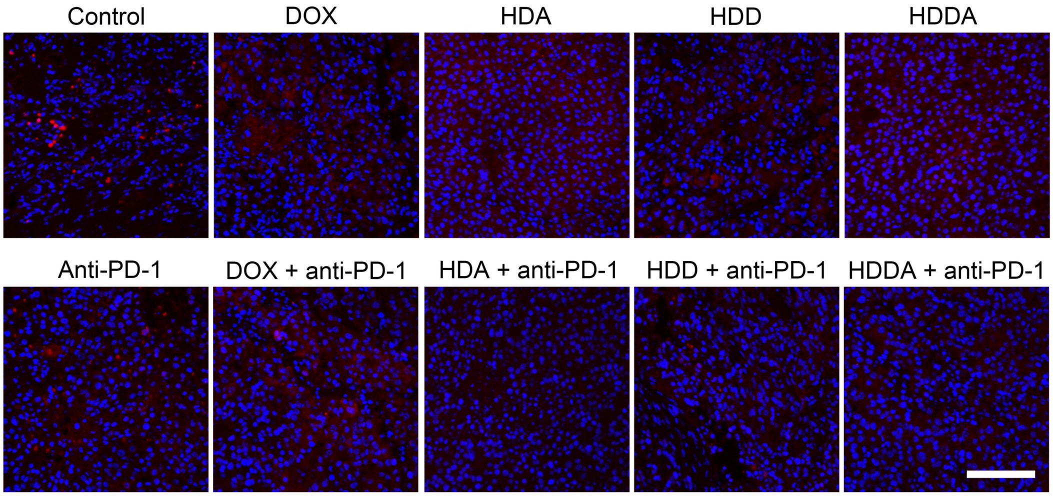

Supplementary Figure 58. Confocal fluorescence images of the representative immunofluorescence staining results of FoxP3 of the tumor tissue slices from the B16F10

tumor-bearing C57BL/6 mice sacrificed at day 7 after different treatments. Scale bar: 100 μm. n = 3 mice per group. The experiment was performed twice with similar results.

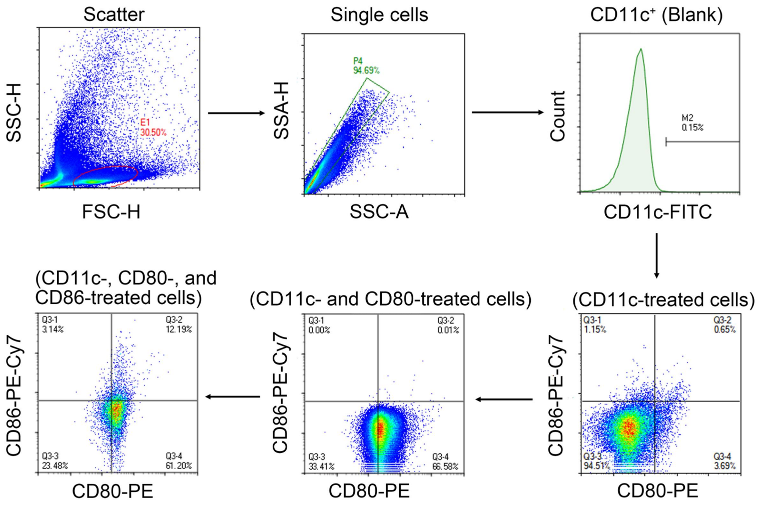

Supplementary Figure 59. Representative flow cytometry gating strategy for CD11c$^+$CD80$^+$CD86$^+$ matured DCs. The experiment was performed twice with similar results.

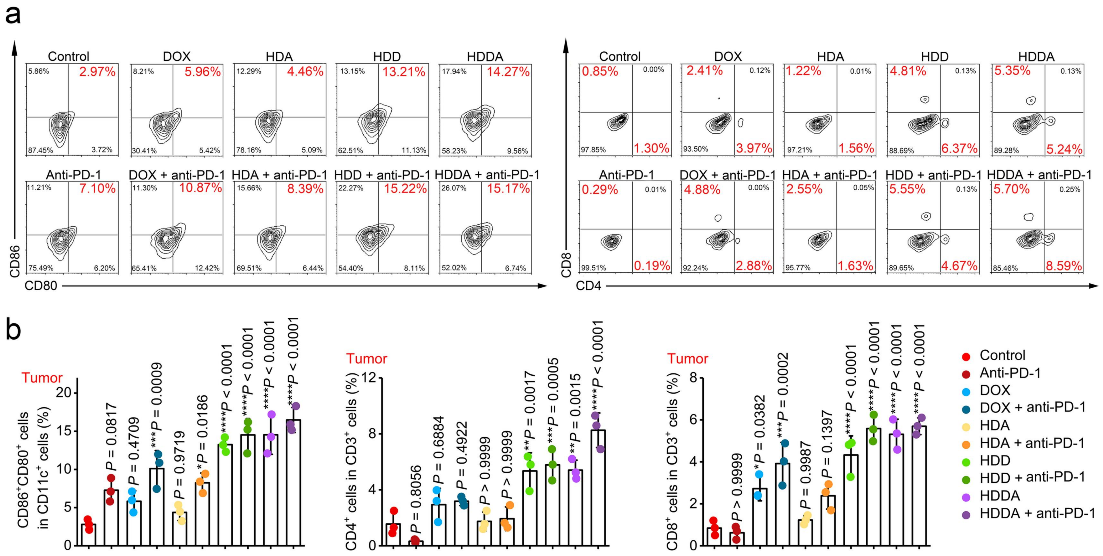

Supplementary Figure 60. Representative flow cytometric plots (**a**) and corresponding quantification results (**b**) of matured DCs (CD11c$^+$CD86$^+$CD80$^+$), cytotoxic T cells (CD3$^+$CD8$^+$), and helper T cells (CD3$^+$CD4$^+$) in the B16F10 tumors retrieved from B16F10-bearing C57BL/6 mice 7 d after different treatments as indicated. Data are presented

as mean ± SD. $n = 3$ mice per group. $^{*}P < 0.05$, $^{**}P < 0.01$, $^{***}P < 0.001$, $^{****}P < 0.0001$. Statistical significance in (**b**) was calculated via one-way ANOVA with a Tukey's post-hoc test.

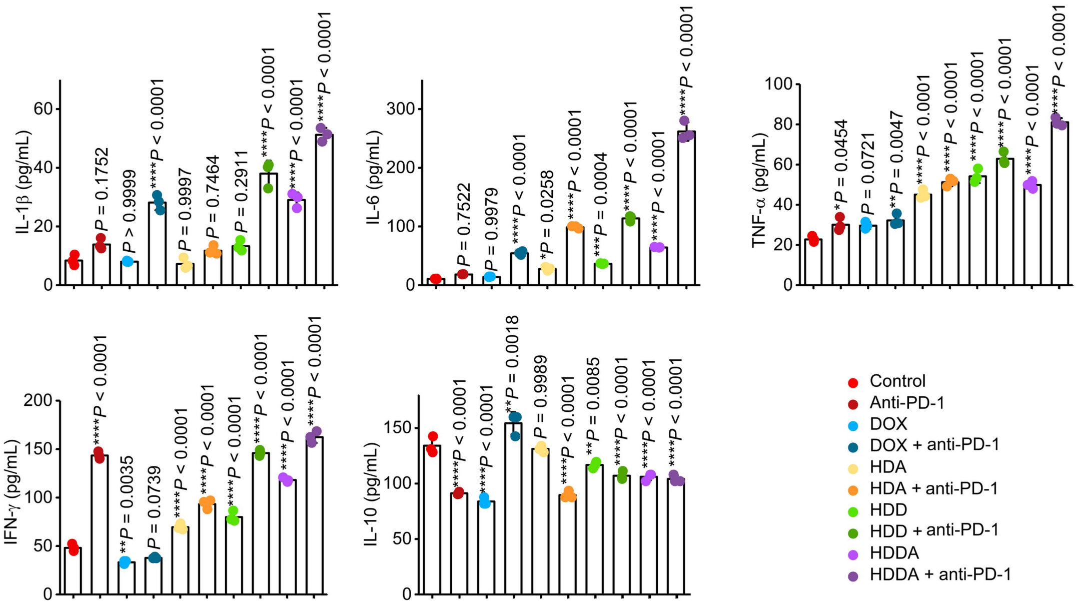

Supplementary Figure 61. ELISA analysis results of the intratumoral expression levels of IL-1β, IL-6, TNF-α, IFN-γ, and IL-10 in the B16F10 tumors retrieved from C57BL/6 mice 3 d after different treatments. Data are presented as mean ± SD. $n = 3$ mice per group. $^{*}P < 0.05$, $^{**}P < 0.01$, $^{***}P < 0.001$, $^{****}P < 0.0001$. Statistical significance was calculated via one-way ANOVA with a Tukey's post-hoc test.

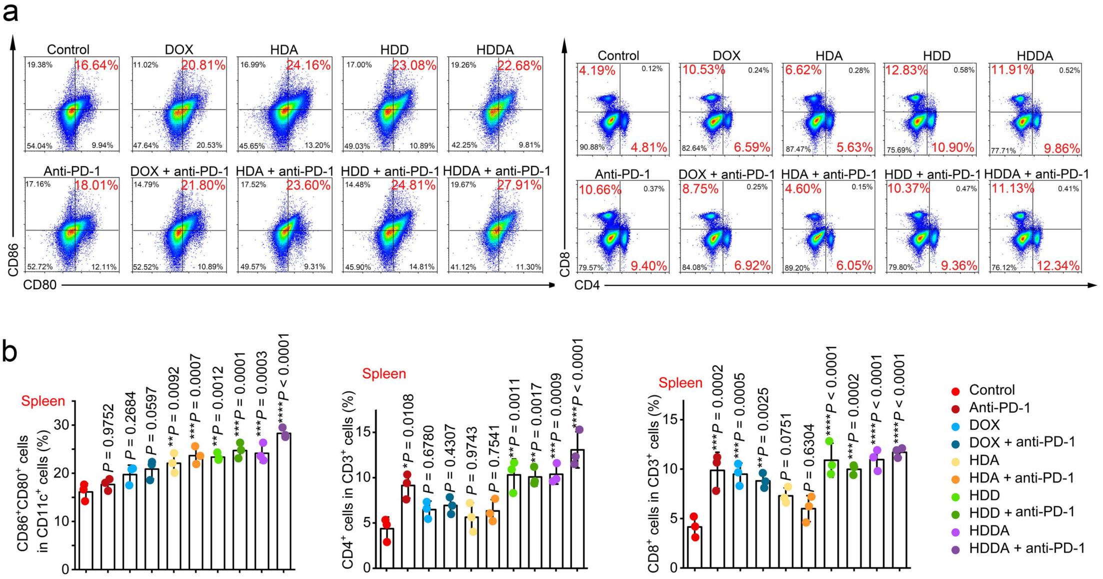

Supplementary Figure 62. Representative flow cytometric plots (**a**) and quantification results (**b**) of matured DCs ($CD11c^+CD86^+CD80^+$), cytotoxic T cells ($CD3^+CD8^+$), and helper T cells ($CD3^+CD4^+$) in the spleens retrieved from B16F10-bearing C57BL/6 mice 7 d after different treatments as indicated. Data are presented as mean ± SD. n = 3 mice per group. *P < 0.05, **P < 0.01, ***P < 0.001, ****P < 0.0001. Statistical significance in (**b**) was calculated via one-way ANOVA with a Tukey's post-hoc test.

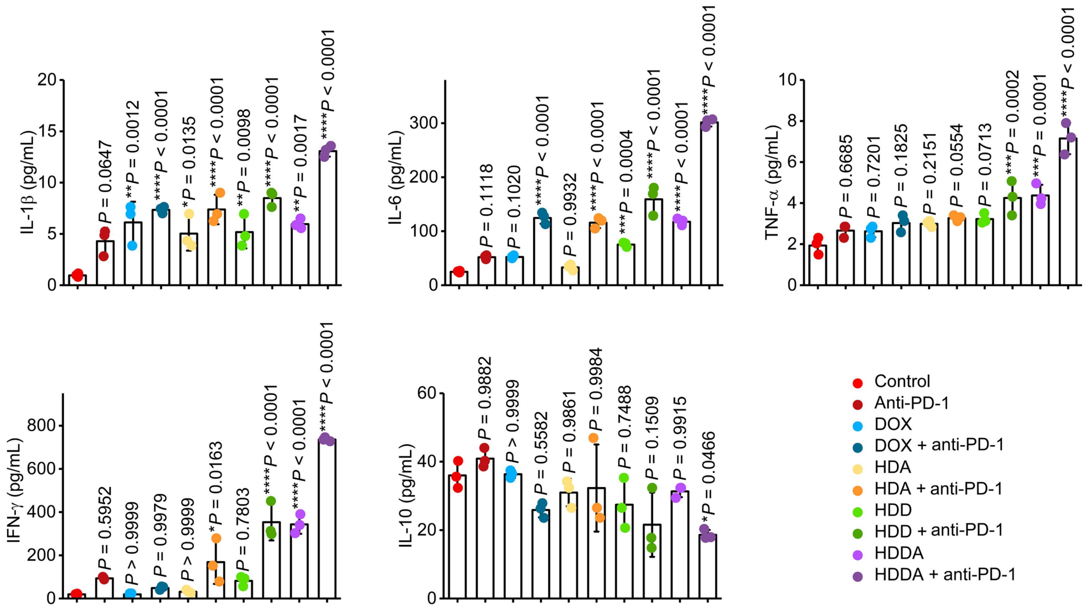

Supplementary Figure 63. ELISA assay results showing the cytokine concentrations of IL-1β, IL-6, TNF-α, IFN-γ, and IL-10 in serum (collected from the B16F10 tumor-bearing

C57BL/6 mice 2 d after different treatments). Data are presented as mean ± SD. $n = 3$ mice per group. *$P < 0.05$, **$P < 0.01$, ***$P < 0.001$, ****$P < 0.0001$. Statistical significance was calculated via one-way ANOVA with a Tukey's post-hoc test.

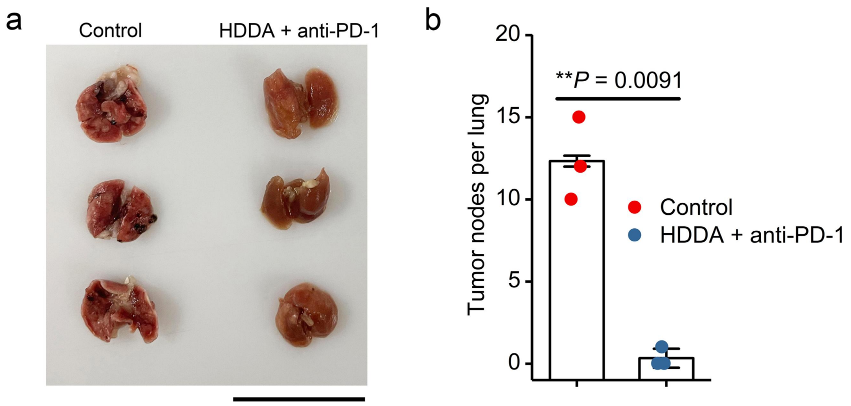

Supplementary Figure 64. Photograph of the lungs (the black regions indicate the tumor nodules) (**a**) and quantification results of the tumor nodules in the lungs (**b**) retrieved from the C57BL/6 mice and "HDDA + anti-PD-1"-healed B16F10 tumor-bearing C57BL/6 mice after intravenous rechallenge of B16F10 cells. **$P < 0.01$. Scale bar: 3 cm. Data are presented as mean ± SD. $n = 3$ mice per group. Statistical significance in (**b**) was calculated via two-tailed Student's t-test.

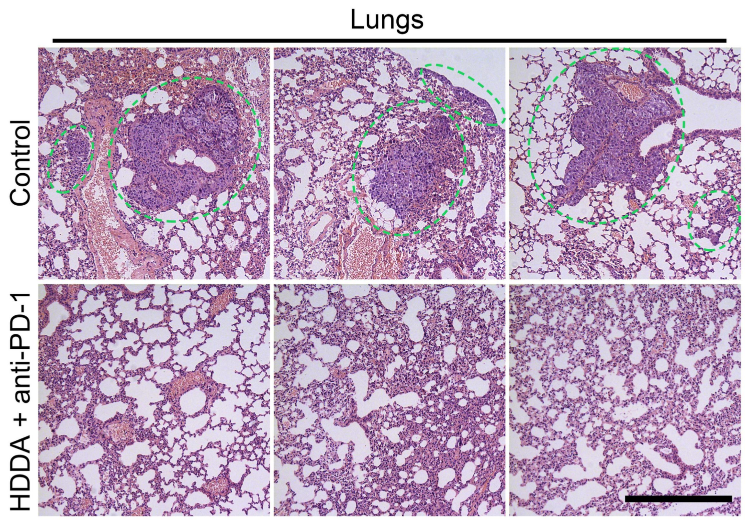

Supplementary Figure 65. H&E-stained tissue slices of the lungs excised from the C57BL/6 mice or "HDDA + anti-PD-1"-healed B16F10 tumor-bearing C57BL/6 mice after intravenous rechallenge of B16F10 cells. Scale bar: 400 μm. The green dotted circles indicate the

positions of tumor nodules. n = 3 mice per group.

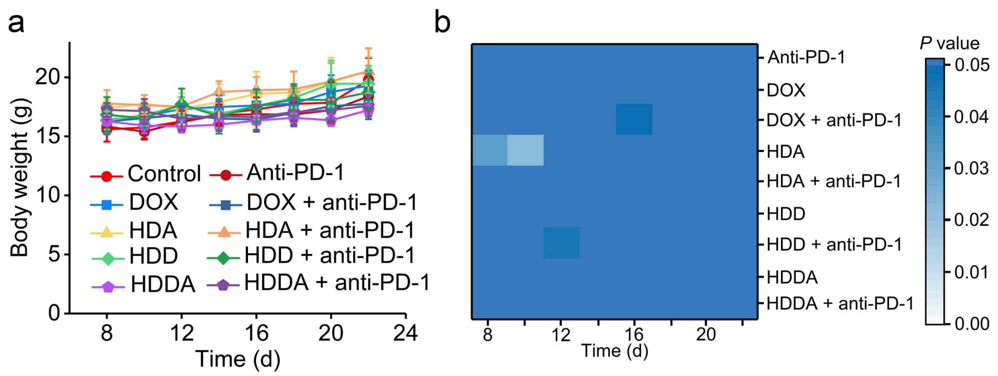

Supplementary Figure 66. (**a**) Body weight changes of the B16F10-bearing C57BL/6 mice after various treatments as indicated (n = 5/group), and (**b**) corresponding heat map of the P values between the control group and other groups. The mice treated with PBS were set as the control group. Data are presented as mean ± SD. Statistical significance in (**b**) was calculated via one-way ANOVA with a Tukey's post-hoc test.

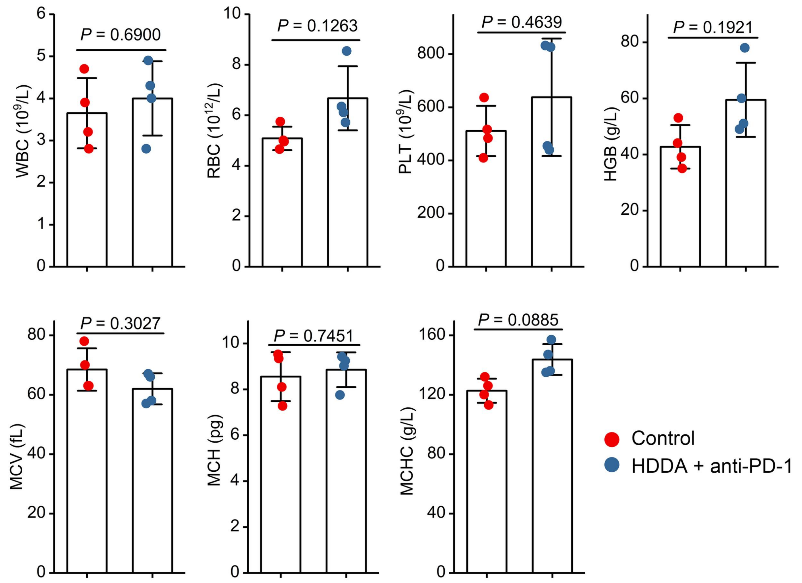

Supplementary Figure 67. Routine blood analysis results of the C57BL/6 mice collected on the 7th day after treatment with PBS (control) or HDDA + anti-PD-1 (DOX dose: 5 mg/kg; n = 4/group). Data are presented as mean ± SD. The blood indexes including WBC, RBC, PLT, HGB, MCV, MCH, and MCHC indicate the numbers of white blood cells, red blood cells, and platelets, concentration of hemoglobin, mean corpuscular volume, mean corpuscular

hemoglobin, and mean corpuscular hemoglobin concentration, respectively. Statistical significance was calculated via two-tailed Student's *t*-test.

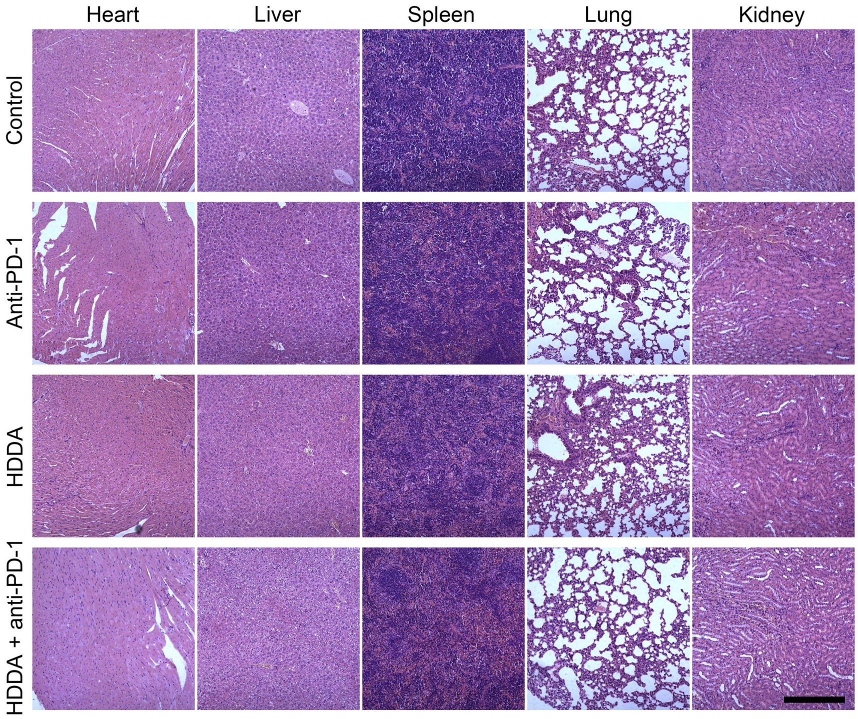

Supplementary Figure 68. H&E-stained tissue slices of the major organs (hearts, livers, spleens, lungs, and kidneys) excised from the C57BL/6 mice collected on the 7th day after treatment with PBS (control), anti-PD-1, HDDA (DOX dose: 5 mg/kg), or HDDA + anti-PD-1 (DOX dose: 5 mg/kg). Scale bar: 300 μm. n = 3 mice per group. The experiment was performed twice with similar results.

Source Data

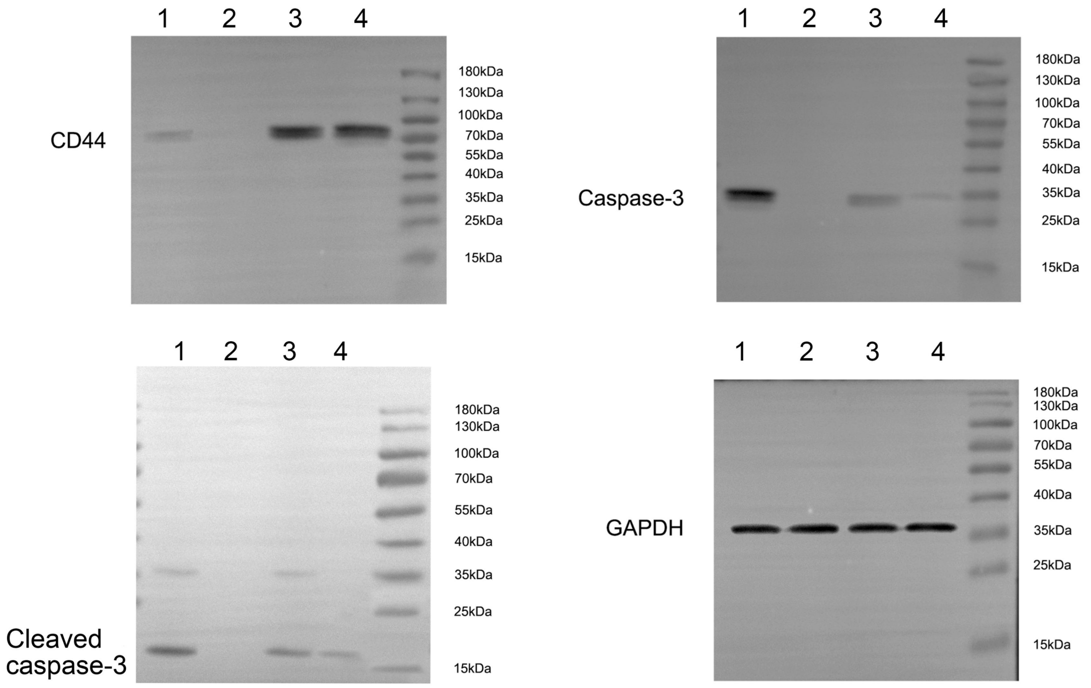

Supplementary Figure 69. The uncropped scans of all gels in Supplementary Fig. 17a. 1, 2, 3, and 4 represent 4T1 cells, cell membranes (4T1), cytoplasm (4T1), and HMVs that were collected from 4T1 cells, respectively.
