# Supplementary material for: In situ generation of micrometer-sized tumor cell-derived vesicles as autologous cancer vaccines for boosting systemic immune responses
Source: Nat Commun. 2022 Nov 1;13:6534. doi: 10.1038/s41467-022-33831-7 (PMC9626595; doi:10.1038/s41467-022-33831-7)
Supplement: Supplementary file 3 — Description of Additional Supplementary Files [file 41467_2022_33831_MOESM3_ESM.pdf]

Supplementary Data 1. Names and functions of the proteins shown in Supplementary Fig. 22a. Protein functions were provided by Applied Protein Technology (Shanghai, China).

Supplementary Data 2. Names and functions of the proteins shown in Supplementary Fig. 22b. Protein functions were provided by Applied Protein Technology (Shanghai, China).

Supplementary Data 3. Gene names and relative expression levels of the selected DEGs in Fig. 6b.
