# Supplementary material for: In situ generation of micrometer-sized tumor cell-derived vesicles as autologous cancer vaccines for boosting systemic immune responses
Source: Nat Commun. 2022 Nov 1;13:6534. doi: 10.1038/s41467-022-33831-7 (PMC9626595; doi:10.1038/s41467-022-33831-7)
Supplement: Supplementary file 7 — Reporting Summary [file 41467_2022_33831_MOESM7_ESM.pdf]

nature portfolio

Corresponding author(s): Fu-Gen Wu

Last updated by author(s): Sep 22, 2022

Reporting Summary

Nature Portfolio wishes to improve the reproducibility of the work that we publish. This form provides structure for consistency and transparency in reporting. For further information on Nature Portfolio policies, see our Editorial Policies and the Editorial Policy Checklist.

Statistics

For all statistical analyses, confirm that the following items are present in the figure legend, table legend, main text, or Methods section.

n/a	Confirmed	
☐	☒	The exact sample size (*n*) for each experimental group/condition, given as a discrete number and unit of measurement
☐	☒	A statement on whether measurements were taken from distinct samples or whether the same sample was measured repeatedly
☐	☒	The statistical test(s) used AND whether they are one- or two-sided<br>*Only common tests should be described solely by name; describe more complex techniques in the Methods section.*
☐	☒	A description of all covariates tested
☐	☒	A description of any assumptions or corrections, such as tests of normality and adjustment for multiple comparisons
☐	☒	A full description of the statistical parameters including central tendency (e.g. means) or other basic estimates (e.g. regression coefficient) AND variation (e.g. standard deviation) or associated estimates of uncertainty (e.g. confidence intervals)
☐	☒	For null hypothesis testing, the test statistic (e.g. *F*, *t*, *r*) with confidence intervals, effect sizes, degrees of freedom and *P* value noted<br>*Give P values as exact values whenever suitable.*
☒	☐	For Bayesian analysis, information on the choice of priors and Markov chain Monte Carlo settings
☒	☐	For hierarchical and complex designs, identification of the appropriate level for tests and full reporting of outcomes
☐	☒	Estimates of effect sizes (e.g. Cohen's *d*, Pearson's *r*), indicating how they were calculated

Our web collection on statistics for biologists contains articles on many of the points above.

Software and code

Policy information about availability of computer code

Data collection	No software was used.
Data analysis	All statistical analyses were performed on GraphPad Prism 9, Excel 2016, or Excel 2019. The flow cytometry data were processed using NovoExpress (version 1.5.0., Agilent).

For manuscripts utilizing custom algorithms or software that are central to the research but not yet described in published literature, software must be made available to editors and reviewers. We strongly encourage code deposition in a community repository (e.g. GitHub). See the Nature Portfolio guidelines for submitting code & software for further information.

Data

Policy information about availability of data

All manuscripts must include a data availability statement. This statement should provide the following information, where applicable:

- Accession codes, unique identifiers, or web links for publicly available datasets
- A description of any restrictions on data availability
- For clinical datasets or third party data, please ensure that the statement adheres to our policy

The main data supporting the results in this study are available within the paper and the supplementary information. All the raw data used to make the figures in this study are available from figshare with the identifier (https://doi.org/10.6084/m9.figshare.20267598.v2). The sequencing data of the transcriptomic analyses in this study are available from the Sequence Read Archive (SRA) Run Selector of the National Center Biotechnology Information (NCBI) database (https://www.ncbi.nlm.nih.gov/Traces/study/) with the NCBI BioProject accession number: PRJNA798199 or the identifier https://www.ncbi.nlm.nih.gov/Traces/study/?acc=PRJNA798199 (for the transcriptomic analysis of 4T1 tumors) and the NCBI BioProject accession number: PRJNA798200 or the identifier https://www.ncbi.nlm.nih.gov/Traces/study/?acc=PRJNA798199 (for the transcriptomic analysis of 4T1 cells). The mass spectrometry proteomics data have been deposited

to the ProteomeXchange Consortium (http://proteomecentral.proteomexchange.org) via the iProX partner repository with the dataset identifier PXD035315. Source data are provided with this paper.

Field-specific reporting

Please select the one below that is the best fit for your research. If you are not sure, read the appropriate sections before making your selection.

- [x] Life sciences
- [ ] Behavioural & social sciences
- [ ] Ecological, evolutionary & environmental sciences

For a reference copy of the document with all sections, see nature.com/documents/nr-reporting-summary-flat.pdf

Life sciences study design

All studies must disclose on these points even when the disclosure is negative.

Sample size	We used G*power analysis to calculate and ensure the sample sizes fulfill adequate power (p > 0.8). According to the experimental data and sample size (n), P value and effect size were calculated and the power was then calculated. If it is more than 80%, demonstrating the sample size is adequate.
Data exclusions	No data were excluded.
Replication	Experiments were repeated and experimental findings were reproducible. Details of experimental replicates were given in the figure legends. All reported attempts at replication were successful.
Randomization	All experimental samples or models were allocated randomly to each group.
Blinding	All the investigators were blinded to group allocation during data collection and analysis.

Reporting for specific materials, systems and methods

We require information from authors about some types of materials, experimental systems and methods used in many studies. Here, indicate whether each material, system or method listed is relevant to your study. If you are not sure if a list item applies to your research, read the appropriate section before selecting a response.

Materials & experimental systems

n/a	Involved in the study	
☐	☒	Antibodies
☐	☒	Eukaryotic cell lines
☒	☐	Palaeontology and archaeology
☐	☒	Animals and other organisms
☒	☐	Human research participants
☒	☐	Clinical data
☒	☐	Dual use research of concern

Methods

n/a	Involved in the study	
☒	☐	ChIP-seq
☐	☒	Flow cytometry
☒	☐	MRI-based neuroimaging

Antibodies

Antibodies used	FITC-labeled goat anti-rabbit IgG antibody (cat. no. bs-0296G-FITC) and anti-mouse CD44-FITC antibody (cat. no. bs-0521R-FITC) were obtained from Bioss Antibodies (Beijing, China). The HRP-labeled goat antirabbit IgG (H + L) antibody (cat. no. E-AB-1003) was bought from Elabscience Biotechnology Co., Ltd. (Wuhan, China). Anti-mouse CD44 (cat. no. mAb #3570) and anti-mouse caspase-3 (cat. no. mAb #9668) were bought from Cell Signaling Technology (Beverly, MA, USA). Anti-mouse GAPDH antibody (cat. no. ab8245) was ordered from Abcam (Cambridge, UK). Anti-mouse cleaved caspase-3 (cat. no. Asp175) was obtained from Affinity Biosciences (Jiangsu, China). PD-1 monoclonal antibody (cat. no. BP1046) was obtained from BioXcell (New Hampshire, USA). Anti-mouse CRT (cat. no. 27298-1-ap) was purchased from Proteintech (Wuhan, China). Anti-mouse CD4 (cat. no. GB13064-2), anti-mouse CD8 (cat. no. GB13429), anti-mouse granzyme B (cat. no. GB14092), anti-mouse Gr-1 (cat. no. GB11229), anti-mouse FoxP3 (cat. no. GB11093), anti-mouse CD206 (cat. no. GB13438), and Cy3-labeled goat anti-rabbit IgG antibody (cat. no. GB21303) were obtained from Wuhan Servicebio Technology Co., Ltd. Anti-mouse CD3-PE (cat. no. 12-0032-82), anti-mouse CD4-FITC (cat. no. 11-0041-82), anti-mouse CD8a (CD8)-APC (cat. no. 17-0081-81), anti-mouse CD8-PE-Cy7 (cat. no. 25-0081-81), anti-mouse MCH II-PE (cat. no. 12-5321-82), anti-mouse CD11c-FITC (cat. no. 11-0114-82), anti-mouse CD80-PE (cat. no. 12-0801-82), and anti-mouse CD86-PE-Cy7 (cat. no. 25-0862-82) were bought from Invitrogen (Carlsbad, USA). Anti-mouse CD11c-PE (cat. no. 117308) and anti-mouse F4/80-FITC (cat. no.124611) were purchased from Biolegend (San Diego, USA). Unless otherwise mentioned, all the antibodies are diluted 200 times before use.
Validation	All antibodies were verified by the supplier and have been quality tested. All validation statements can be found in the respective antibody website:<br>1. Anti-mouse PD-1: https://bxcell.com/product/m-cd279/<br>2. Anti-mouse CD86-PE-Cy7: https://www.thermofisher.cn/cn/zh/antibody/product/CD86-B7-2-Antibody-clone-GL1-

Monoclonal/25-0862-82
3. Anti-mouse CD80-PE: https://www.thermofisher.cn/cn/zh/antibody/product/CD80-B7-1-Antibody-clone-16-10A1-Monoclonal/12-0801-82
4. Anti-mouse CD11c-FITC: https://www.thermofisher.cn/cn/zh/antibody/product/CD11c-Antibody-clone-N418-Monoclonal/11-0114-82
5. Anti-mouse MCH II-PE: https://www.thermofisher.cn/cn/zh/antibody/product/MHC-Class-II-I-A-I-E-Antibody-clone-M5-114-15-2-Monoclonal/12-5321-82
6. Anti-mouse CD8a-APC: https://www.thermofisher.cn/cn/zh/antibody/product/CD8a-Antibody-clone-53-6-7-Monoclonal/17-0081-81
7. Anti-mouse CD4-FITC: https://www.thermofisher.cn/cn/zh/antibody/product/CD4-Antibody-clone-GK1-5-Monoclonal/11-0041-82
8. Anti-mouse CD3-PE: https://www.thermofisher.cn/cn/zh/antibody/product/CD3-Antibody-clone-17A2-Monoclonal/12-0032-82
9. Anti-mouse CD11c-PE: https://www.biolegend.com/en-us/products/pe-anti-mouse-cd11c-antibody-1816
10. Anti-mouse F4/80-FITC: https://www.biolegend.com/en-us/search-results/fitc-anti-mouse-f4-80-antibody-4067
11. Anti-mouse CD4: https://www.servicebio.cn/goodsdetail?id=4459
12. Anti-mouse CD8: https://www.servicebio.cn/goodsdetail?id=4312
13. Anti-mouse granzyme B: https://www.servicebio.cn/goodsdetail?id=354
14. Anti-mouse Gr-1: https://www.servicebio.cn/goodsdetail?id=1454
15. Anti-mouse FoxP3: https://www.servicebio.cn/goodsdetail?id=1373
16. Anti-mouse CD206: https://www.servicebio.cn/goodsdetail?id=4315
17. Cy3-labeled goat anti-rabbit IgG antibody： https://www.servicebio.cn/goodsdetail?id=253
18. Anti-mouse CRT: https://www.ptgcn.com/products/Calreticulin-Antibody-27298-1-AP.htm
19. FITC-labeled goat anti-rabbit IgG antibody: http://www.bioss.com.cn/prolook_03.asp?id=AF08169606011198&pro37=4
20. Anti-mouse CD44-FITC: http://www.bioss.com.cn/prolook_03_biaoji.asp?id=AF08169600608930&pro37=2&pro33=206
21. HRP-labeled goat antirabbit IgG (H + L) antibody: https://www.elabscience.cn/p-goat_anti_rabbit_igg_h_l_peroxidase_hrp_conjugated_-77928.html
22. Anti-mouse CD44: https://www.cellsignal.cn/products/primary-antibodies/cd44-156-3c11-mouse-mab/3570?site-search-type=Products&N=4294956287&Ntt=anti-mouse+cd44&fromPage=plp
23. Anti-mouse caspase-3: https://www.cellsignal.cn/products/primary-antibodies/caspase-3-3g2-mouse-mab/9668?site-search-type=Products&N=4294956287&Ntt=anti-mouse+caspase-3&fromPage=plp
24. Anti-mouse cleaved caspase-3: http://www.affbiotech.cn/goods-2074-AF7022-Cleaved_Caspase_3_Asp175_p17_Antibody.html
25. Anti-mouse GAPDH: https://www.abcam.cn/gapdh-antibody-6c5-loading-control-ab8245.html
26. Anti-mouse CD8-PE-Cy7: https://www.thermofisher.cn/cn/zh/antibody/product/CD8a-Antibody-clone-53-6-7-Monoclonal/25-0081-81

Eukaryotic cell lines

Policy information about cell lines

Cell line source(s)	4T1, B16F10, MCF-7, MCF-7/ADR, NIH 3T3, and RAW 264.7 cells were obtained from KeyGEN BioTECH, China. HMEC-1, HPAEpiC, and MDA-MB-231 cell line were purchased from ATCC, USA. Primary bone marrow-derived dendritic cell (BMDC) cells were prepared and cultured by following a standard protocol.
Authentication	Identity of the cell lines were frequently checked by their morphological features but have not been authenticated by the short tandem repeat (STR) profiling.
Mycoplasma contamination	All cell lines were tested for mycoplasma contamination. No mycoplasma contamination was found.
Commonly misidentified lines (See ICLAC register)	No commonly misidentified cell lines were used in this study.

Animals and other organisms

Policy information about studies involving animals; ARRIVE guidelines recommended for reporting animal research

Laboratory animals	BALB/c and C57BL/6 mice (female, 6–8 weeks) were ordered from Yangzhou University Medical Center (Yangzhou, China). Mice were housed in groups of 5 mice per individually ventilated cage with constant room temperature (25 ± 3 °C) with 12 h dark–light cycles and a relative humidity of 40–70%. All mice had access to food and water ad libitum.
Wild animals	The study did not involve wild animals.
Field-collected samples	The study did not involve samples collected from the field.
Ethics oversight	All animal experiments were performed in accordance with the permission from the ethics committee of Southeast University, China (No. 20211224002). All experiments were performed in compliance with the Regulations for the Administration of Affairs Concerning Experimental Animals of China. All experiments followed institutional guidelines.

Note that full information on the approval of the study protocol must also be provided in the manuscript.

Flow Cytometry

Plots

Confirm that:

- [x] The axis labels state the marker and fluorochrome used (e.g. CD4-FITC).
- [x] The axis scales are clearly visible. Include numbers along axes only for bottom left plot of group (a 'group' is an analysis of identical markers).
- [x] All plots are contour plots with outliers or pseudocolor plots.
- [x] A numerical value for number of cells or percentage (with statistics) is provided.

Methodology

Sample preparation	The tissue samples were passed through 200-mesh nylon mesh filters to obtain single-cell suspensions.<br><br>For all samples, cells were first stained with antibodies against surface antigens. In some experiments, cells were subsequently fixed, permeablized, and stained for intracellular antigens.<br><br>The detailed sample preparation method could be found in the article file and the supplementary information.
Instrument	NovoCyte 2070R, ACEA Biosciences Inc., USA
Software	NovoExpress (version 1.5.0., Agilent)
Cell population abundance	No sorting was performed.
Gating strategy	Generally, cells were first gated on FSC/SSC. Singlet cells were gated using SSC-H and SSC-A. Dead cells were excluded and surface and intracellular antigen gating was performed on the singlet cell population. The cell populations were then analyzed based on the expression of markers. Gating was then based on positive level. The detailed gating strategy could be found in the supplementary information.

- [x] Tick this box to confirm that a figure exemplifying the gating strategy is provided in the Supplementary Information.
